# Glutamine Metabolism: Molecular Regulation, Biological Functions, and Diseases

**DOI:** 10.1002/mco2.70120

**Published:** 2025-06-25

**Authors:** Mudasir A. Kumar, Sana Khurshid Baba, Inamu Rashid Khan, Mohd Shahnawaz Khan, Fohad Mabood Husain, Saheem Ahmad, Mohammad Haris, Mayank Singh, Ammira S. Al‐Shabeeb Akil, Muzafar A. Macha, Ajaz A. Bhat

**Affiliations:** ^1^ Watson‐Crick Centre for Molecular Medicine Islamic University of Science and Technology Kashmir India; ^2^ Department of Zoology School of Life Sciences Central University of Kashmir Ganderbal India; ^3^ Department of Biochemistry College of Sciences King Saud University Riyadh Saudi Arabia; ^4^ Department of Food Science and Nutrition College of Food and Agriculture Sciences King Saud University Riyadh Saudi Arabia; ^5^ Department of Medical Laboratory Sciences College of Applied Medical Sciences University of Hail Hail City Saudi Arabia; ^6^ Center for Advanced Metabolic Imaging in Precision Medicine Department of Radiology Perelman School of Medicine University of Pennsylvania Philadelphia USA; ^7^ Laboratory Animal Research Centre Qatar University Doha Qatar; ^8^ Department of Medical Oncology (Lab.) Dr. BRAIRCH All India Institute of Medical Sciences (AIIMS) New Delhi India; ^9^ Department of Human Genetics‐Precision Medicine in Diabetes Obesity and Cancer Program, Sidra Medicine Doha Qatar

**Keywords:** cancer, circular RNAs, glutaminolysis, glutamine metabolism, long noncoding RNAs, therapeutic targeting

## Abstract

Glutaminolysis, the metabolic process of converting glutamine into key intermediates, plays an essential role in cellular energy production, signaling, biosynthesis, and redox balance. Deregulation of glutamine metabolism significantly influences various pathological conditions, including cancers and metabolic and neurological diseases. Emerging evidence shows that long noncoding RNAs (lncRNAs), circular RNAs (circRNAs), and oncogenic alterations in glutamine transporters and enzymes enhance glutamine's role as an alternative energy source, supporting cell survival and proliferation under nutrient and oxygen deprivation conditions. To combat the pathogenic effects of altered glutamine metabolism, researchers are developing targeted inhibitors of key enzymes and transporters involved in glutaminolysis. By interfering with the mechanisms that support the growth of cancer cells, these inhibitors may be able to stop the growth of tumors and treat metabolic and neurological conditions. This review provides a comprehensive overview of existing inhibitors and ongoing clinical trials targeting glutamine metabolism, focusing on its potential as a cancer therapeutic strategy. Additionally, the role of lncRNAs and circRNAs in regulating glutamine metabolism is explored, revealing novel avenues for therapeutic intervention in cancer and other diseases.

## Graphical abstract

Tumor cells adapt to nutrient‐poor environments by altering metabolism to acquire essential nutrients. They convert glutamine into glutamate and α‐ketoglutarate, supporting mTOR activation and sugar biosynthesis. Disruption of mTORC1 signaling is linked to disease, while glutamine and leucine activation promotes cell growth and inhibits autophagy. Glutamine inhibitors are being explored for treatment.

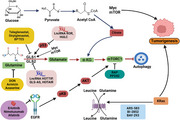

## 1 Introduction

The intricate relationship between altered metabolism and disease has become central to biomedical research, particularly cancer biology. Among the hallmark traits of cancer is metabolic reprogramming, which allows tumor cells to thrive in nutrient‐deprived environments. One of the pivotal elements of this reprogramming is the dependency on external resources provided by the tumor microenvironment (TME) [1]. Cancer cells adapt to the harsh conditions within the TME by modifying their metabolic pathways to support rapid proliferation, evade immune responses, and resist treatment. These adaptations primarily involve changes in the metabolism of carbohydrates, lipids, and amino acids‐key drivers of cellular growth and survival [2]. Glucose, glutamine, and leucine are vital nutrients in nutrient‐poor microenvironments that provide energy and generate building blocks such as nucleotides, proteins, and lipids. Glucose and glutamine, two critical nutrients, are indispensable substrates in tumor cell metabolism, supporting bioenergetics and biosynthesis [3, 4, 5]. Glutamine, the most abundant amino acid in the body, plays a crucial role in these processes. Cancer cells exhibit a marked dependence on glutamine to fuel anaplerosis, generate metabolic intermediates, and maintain redox balance. This “glutamine addiction” is not limited to providing energy but extends to supporting the biosynthesis of nucleotides, proteins, and lipids, all essential for the uncontrolled proliferation of cancer cells [6, 7]. Besides serving as a fuel source, glutamine is involved in cytoprotective programs that protect cancer cells against harmful agents in the TME [8, 9]. Cancer cells metabolize glutamine through glutaminolysis within the mitochondria, converting it to glutamate and tricarboxylic acid cycle (TCA) intermediary α‐ketoglutarate (αKG) [10].

The diversion of pyruvate from the TCA cycle leads to increased dependence on glutamine as a carbon source for anaplerosis [11, 12]. Glutamine is transported into the cell by solute carrier (SLC) type transporters and is then catabolized by the enzyme glutaminase (GLS1), which converts glutamine into glutamate and ammonia. αKG is then produced by the metabolism of glutamate by transaminases or glutamate dehydrogenase (GLUD) [13]. αKG then undergoes carboxylation to produce isocitrate catalyzed by aconitase to produce citrate. Isocitrate is converted to citrate through aconitase reverse reaction, allowing carbon for ATP citrate lyase to produce acetyl CoA. This process enables ATP production and provides necessary biosynthetic precursors for cancer cell growth and proliferation [14, 15, 16]. This heightened demand for glutamine underscores its significance in cancer and other diseases characterized by metabolic dysregulation. Over the years, research into glutamine metabolism has uncovered its broad influence on various biological processes, including cell signaling, autophagy, and apoptosis [17, 18, 19, 20]. These findings have positioned glutamine metabolism as a potential therapeutic target across multiple diseases.

The central role of glutamine metabolism in diseases has prompted research exploring its molecular regulation and biological functions. Recent studies have shed light on how glutamine metabolism is controlled at the molecular level, revealing key regulators such as oncogenes and tumor suppressors that orchestrate its uptake and utilization in cancer cells. Additionally, the discovery of glutaminase inhibitors and other metabolic modulators has opened new avenues for therapeutic intervention, particularly in targeting the vulnerabilities of cancer cells’ reliance on glutamine. However, despite these advances, significant gaps remain in our understanding of how glutamine metabolism interacts with other metabolic pathways and contributes to disease progression.

This review aims to provide a comprehensive overview of glutamine metabolism, focusing on its regulation, functions, and implications in various diseases. We first explored the historical background and current state of research on glutamine metabolism. Then, we searched the molecular mechanisms governing glutamine uptake, transport, and utilization, particularly in cancer. Finally, we highlighted emerging therapeutic strategies targeting glutamine metabolism, emphasizing the potential for future clinical applications. By synthesizing these findings, this review provides insights into the critical role of glutamine metabolism and its therapeutic potential in disease management.

## 2 Role of Glutaminolysis in Cancer

Cancer metabolism has gained interest for nearly a century due to its ability to uncover fundamental aspects of malignancy and its potential to improve cancer diagnosis, monitoring, and treatment. Glutamine metabolism, the most abundant amino acid in plasma, is crucial in cancer due to its ability to donate nitrogen and carbon into growth‐promoting pathways. During periods of rapid growth or stress, glutamine becomes conditionally essential, especially in cancer cells that display oncogene‐dependent addictions. Many cancer cells rely heavily on glutamine for survival, a phenomenon known as glutamine dependence. This underscores glutamine's essential role in their ability to thrive. Cancer cells adapt to support the citric acid cycle by increasing glutamine metabolism, which is crucial for their growth. Glutamine provides carbon for the cycle and supplies nitrogen needed to synthesize hexosamines, nucleotides, and various nonessential amino acids [21, 22].

In cancers like non‐small cell lung cancer (NSCLC), brain tumors, and breast cancer (BC), glutamine metabolism is a vital process. Additionally, it influences the TME by regulating oxidative stress through glutathione (GSH) production [23]. Glutamine metabolism regulators, including amino acid transporters SLC1A5, SLC7A5, SLC7A11, SLC3A2, and Myc, are critical for maintaining the balance between glutamine metabolism and cell viability [24, 25]. Increased expression of glutamine metabolism regulators has been linked to a high survival rate in multiple cancers. Tumor cells upregulate most glycolytic enzymes due to increased c‐Myc and hypoxia‐inducible factor 1 alpha (HIF‐1α) transcriptional activity, insufficient control by p53 and other tumor suppressors, and oncogenes such as mutant Kirsten rat sarcoma virus (KRAS). These factors induce glutaminolysis by directly or indirectly activating glutamine transporters and glycolytic genes in cancer cells [3]. Glutamine and its metabolites play crucial roles in various cellular mechanisms, including mTOR activation and the biosynthesis of sugars, nucleic acids, amino acids, and fatty acids [9]. Dysregulation of the mTORC1 signaling pathway is associated with pathological conditions, including cancer, obesity, diabetes, and neurodegeneration [26]. In certain types of cancer, overexpression of mTORC1 occurs due to Tuberous sclerosis protein 1/2 (TSC) phosphorylation and inactivation by Cyclin D1–CDK4/6. Glutamine flux has been reported to modulate mTOR to coordinate cell proliferation and growth [27]. Recent studies have indicated that mTORC1, a signaling pathway involved in cellular metabolism, regulates aerobic glycolysis through HIF‐1α. Recent studies have found new functions for glutamine in regulating cell proliferative events. For example, cancer cells under hypoxia or with defective mitochondria can use glutamine‐derived α‐KG to produce citrate, which is crucial for lipid synthesis, highlighting the importance of glutamine in cell proliferation. Because of the energy requirements of rapidly proliferating cells, tumors produce a hypoxic environment as they develop. According to current research, mTORC1 regulates aerobic glycolysis using HIF‐1α  lowers hypoxia by inducing angiogenesis and regulates the metabolism of cells [28]. Furthermore, dysregulation of oxidative phosphorylation (OXPHOS) and autophagy are key metabolic characteristics of metastatic tumor cells, which frequently experience metabolic stress [29]. NcRNAs, including miRNAs [30], long noncoding RNAs (lncRNAs) [31], and circular noncoding RNAs (cncRNAs) [32], have been discovered as glutaminolysis regulators, and they may interact with oncogenes and tumor suppressors genes and influence the metabolism of cancer. Research has confirmed the contribution of ncRNAs to cancer progression, impacting crucial cancer signatures such as glutaminolysis. LncRNAs, newly discovered functional noncoding RNAs, exert significant regulatory effects through various mechanisms. Recent studies have demonstrated the extensive function of lncRNAs in controlling many biological functions, such as metabolic processes. Gaining insight into the intricate nature of lncRNAs will help us comprehend tumor metabolic machinery and make it easier to build lncRNA‐based cancer metabolism‐targeting therapeutics. Tumor suppressor genes like p53 or oncogenes like c‐Myc can control lncRNA functions. In contrast, lncRNAs and circular RNAs (circRNAs) can impact the production of HIF‐1α [33] and c‐Myc [34]. The atypical metabolic rewiring observed in tumors is an important cancer characteristic. It has been explored for diagnostic, monitoring, and targeted therapeutic techniques, making it a promising target for cutting‐edge therapies. In addition to cancer, glutaminolysis is an important player in the pathophysiology of several other diseases, including neurological, kidney, autoimmune, and cardiovascular diseases [35, 36, 37]. Recent research on immune metabolism has highlighted the role of glutamine in immune system regulation [38]. It has been observed that glutamine is necessary for immune cells to survive, proliferate, differentiate biologically, and defend against various diseases [39, 40]. Recent research suggests that glutamine is also crucial in cardiovascular physiology and pathology, as it aids in synthesizing DNA, ATP, proteins, and lipids, driving vital vascular cell processes [41]. The dysregulation of glutamate metabolism is responsible for glutamate excitotoxicity [42, 43]. The glutamate excitotoxicity idea holds that excessive glutamate promotes neuronal dysfunction and degeneration [44]. It has important implications for both acute CNS insults, such as ischemia and traumatic brain damage, as well as chronic neurodegenerative illnesses, including amyotrophic lateral sclerosis (ALS), multiple sclerosis, and Parkinson's disease (PD) [45]. Despite continuous research, no pharmaceutical therapies are available to provide considerable neuroprotection in brain ischemia or damage cases. Also, metabolic reprogramming influences the progression and prognosis of kidney diseases. At the same time, glutaminolysis generates ammonia, essential for maintaining renal pH and cellular and systemic homeostasis, and is released from glutamine breakdown [46]. Several different regulators have a distinct role in how this process functions, as seen by the metabolic pathway that breaks down glutamine. Different strategies employed by these regulators can impact the efficiency and results of glutaminolysis. The significance of these activities is especially evident in different diseases, as the disruption of glutamine metabolism can potentially exacerbate the disease's genesis or progression. For example, certain regulators may block the route, which might have therapeutic effects, while others may strengthen it, promoting the fast proliferation of cancer cells. As a result, identifying the major regulators of glutaminolysis is critical for successful targeting. This could be useful in developing strategies to prevent or mitigate the effects of these diseases.

## 3 Altered Metabolic Genes in Glutaminolysis

**FIGURE 1:**
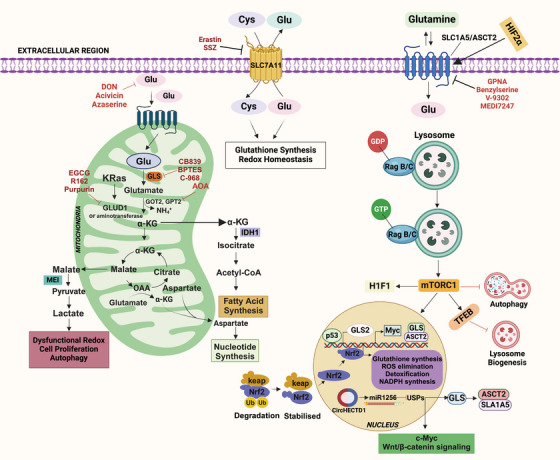
Glutamine signaling pathway and metabolism in cancer. Glutamine enters cells through transporters and cotransporters like SLC1A5/ASCT2 and SLC7A11. It is converted to α‐ketoglutarate (α‐KG), enhancing mTORC1 signaling and HIF‐1α accumulation. mTORC1 regulates ASCT2, GLS, and HIF‐1 via c‐Myc. It inhibits lysosome biogenesis and autophagy. Malate supports nucleotide synthesis and produces pyruvate and NADPH. Mutated Keap1 induces GSH synthesis, ROS elimination, and NADPH synthesis. circHECTD1 targets miR‐1256 to modulate β‐catenin/c‐Myc signaling, activating GLS and ASCT2/SL1A5.

Mutations in metabolic genes related to glutaminolysis can significantly influence cancer metabolism, affecting both cell growth and survival. These genetic alterations often enhance the conversion of glutamine to αKG, which fuels the TCA cycle, promoting energy production and biosynthesis. Tumor suppressors and oncogenes play essential roles in regulating glutamine metabolism, and their activity can profoundly impact the function of glutamine and its metabolites in cancer cells [47] (Figure 1). It has been determined that certain cancer forms contain mutations in metabolic enzymes, such as TP53, retinoblastoma protein (Rb), and HIF‐1α, as well as enzymes like hexokinase (HK), pyruvate kinase isozymes M2 (PKM2), isocitrate dehydrogenase (IDH), succinate dehydrogenase (SDH), and nicotinamide phosphoribosyl transferase (Nampt), CDKN2A, or activating mutations of NFE2L2, NOTCH1/2, MLL2, and EP300 [48] driving tumor progression and metastasis [49, 50]. Also, many oncogenic agents and tumor suppressors directly control the metabolic reprogramming of cancer cells. Myc, MycL, and MycN are among the Myc family members of oncoproteins, the master regulators of metabolic reprogramming in a wide range of human malignancies. In this context, the c‐Myc oncogene plays a pivotal role, particularly in glutamine metabolism. The c‐Myc oncogene acts as a master regulator of cellular growth and metabolism, orchestrating a wide range of metabolic changes in transformed cells to facilitate rapid proliferation. When c‐Myc is overexpressed, it leads to coordinated changes in the expression of various gene families, which collectively enhance cellular growth and division. One significant effect of c‐Myc overexpression is the upregulation of GLS1, an enzyme responsible for converting glutamine into glutamate. In various types of cancers, it enhances the glutamate‐ammonia ligase (GLUL) expression level, which is involved in the fresh synthesis of glutamine. Hyperactivation of c‐Myc has been associated with glutamine addiction [51, 52]. mTORC1 modulates GLS levels via S6K1‐dependent c‐Myc regulation, improving translation efficiency by modifying eIF4B phosphorylation, which is required to unravel the 5′UTR structure [53]. Dysregulation of the mTORC1 signaling pathway is linked to several pathological conditions, including cancer, obesity, diabetes, and neurodegeneration [26]. Phosphorylation and inactivation of TSC1/2 by Cyclin D1–CDK4/6 lead to overexpression of mTORC1 in certain types of cancer [54, 55]. The study of p53's role in regulating the mTOR pathway has gained interest due to its crucial role in tumorigenesis. The coordinated regulation of p53 and the mTOR pathway is essential for maintaining homeostasis in response to stimuli. p53 controls the mTOR pathway at multiple levels, including direct regulating signaling mechanisms, posttranscriptional regulation by miRNAs, and inhibiting autophagy through protein‐protein interaction [56]. p53 plays an essential role in glutamine metabolism by regulating the gene expression of glucose transporters GLUT1 and GLUT4. Elevations in glycolysis and energy availability are caused by polymorphisms in the DNA‐binding domain of p53, which eliminates its suppression of GLUT1 and GLUT4 genes being expressed. p53 upregulated the glutaminase 2 (GLS2) enzyme, leading to increased GSH levels and decreased reactive oxygen species (ROS) levels, safeguarding cells against damage to DNA [57]. In response to oxidative stress or DNA damage, p53 promotes GLS2 synthesis in a p53‐dependent manner, and p53 interacts with the GLS2 promoter. The tumor suppressor p53 regulates the expression of the genes that control mitochondrial oxidative respiration, namely GLS2 and cytochrome *c* oxidase deficient homolog 2 (SCO2). The balance of glucose consumption is shifted from mitochondrial respiration to the anaerobic glycolytic route by altered SCO2. At the same time, overexpression of GLS2 by p53 increases the level of GSH and decreases ROS, finally defending cells against DNA damage [57, 58]. Aberrant expression of GLS1 has been found in hepatocellular carcinoma (HCC), contributing to malignancy and poor prognosis. GLS1 knockdown inhibits the proliferation of cancerous cells in the liver and prevents tumor formation [59, 60, 61]. Overexpression of GLS1 has also been observed in human colorectal cancer (CRC) tissues and NSCLC. Data from the TCGA database reveal overexpression of GLS1 in various solid tumors, including stomach adenocarcinoma, head and neck squamous cell carcinoma, thymoma, testicular germ cell tumors, HCC, colon adenocarcinoma, and others. Overexpression of glutamine transporters (ASCT2) has been observed in various cancers, contributing to increased glutamine uptake and offering it a possible therapeutic target for the control of cancer. c‐Myc and HIF‐1α regulate multiple glycolytic enzymes and proteins involved in glutaminolysis and fatty acid synthesis [62]. HIF‐1α is involved in regulating glutamate transporters and glutamate receptors [63]. A study showed that HIF‐1α stimulates glutamine metabolism in CRC by increasing GLS1 expression and activity [64]. The activation and stabilization of HIF‐1α play a crucial role in cellular metabolic adaptations to hypoxia. Prolyl hydroxylase domain (PHD) proteins are oxygen‐sensing enzymes that hydroxylate HIF‐1α at a proline residue at normal oxygen levels. The ubiquitin ligase von Hippel Lindau (VHL) then degrades the protein. Then, ubiquitin ligase VHL degrades this hydroxylated enzyme [65]. Cancer cells with elevated HIF‐1α levels tend to exhibit higher malignancy and poorer response to radiotherapy, leading to a worse prognosis.

IDH1 and IDH2 isoforms are frequently mutated in various cancers, including glioma, acute myeloid leukemia, thyroid carcinomas, cartilaginous tumors, and intrahepatic cholangiocarcinoma (ICC) [66, 67, 68, 69]. Mutations in IDH result in the accumulation of R‐2‐hydroxyglutarate (R‐2‐HG) [70, 71, 72], which activates PHD, leading to increased prolyl hydroxylation of HIF‐1α and subsequent degradation. Inactivation of SDH, also known as succinate‐coenzyme Q reductase or Complex II, leads to the accumulation of succinate, which inhibits PHDs and causes an increase in HIF‐1α protein levels [73]. Accordingly, the build‐up of R‐2‐HG caused by IDH1/2 mutations decreases HIF‐1α levels and encourages tumor development, which includes astrocyte cancer [74, 75]. Cancer cell metabolic plasticity relies on activating and inhibiting various genes, oncogenes, growth factors, and tumor suppressors. A critical factor in this process is the interaction between HIF‐1α and AMP‐activated protein kinase (AMPK), which serves as an energy sensor and essential regulator of cellular metabolism. Glutaminolysis is critical in ATP production to turn off AMPK and mTORC1 [76]. AMPK activation, triggered by the binding of AMP or ADP, redirects metabolism toward increased catabolism and decreased anabolism by phosphorylating downstream critical protein networks that, in the end, cause mTORC1 suppression [77]. When there are dietary imbalances, glutaminolysis drives mTORC1 activation, which results in abnormal suppression of autophagy and glutaminolysis [78, 79]. Two parallel pathways of glutamine metabolism promote mTORC1 activation: one that relies on glutaminolysis and is mediated by RagB, and the other that is dependent on ATP and not on GLS or glutamine dehydrogenase (GDH) [80].

Metabolic reprogramming in idiopathic pulmonary fibrosis (IPF) lung fibroblasts involves glutaminolysis, contributing to their resistance to apoptosis. Reducing glutamine metabolism induces apoptosis in IPF fibroblasts, leading to changes in antiapoptotic gene expression and various epigenetic processes [81, 82]. Survivin and X‐linked inhibitors of apoptosis protein (XIAP), which belong to the regulator of apoptosis protein (IAP) family, have less expression when glutaminolysis is inhibited [83]. Glutamate plays a role in invasion through the involvement of GLS1 and the metabotropic glutamate receptor GRM3. GRM3 is associated with the growth of malignant brain tumors, including glioma and breast and melanoma cancers [84, 85, 86, 87, 88, 89]. Mutations in GRM3 result in constitutive receptor activation, provide signals for cell proliferation and survival in melanoma [89], and are closely associated with invasive behavior [90]. Mutations in PIK3CA are also related to the glutamine metabolism imbalance, as seen in intestinal cancer, which makes cancer cells rely on glutamine through overactivation of glutamate‐pyruvate transaminase 2 (GPT2) [91]. Dysregulated RAS signaling has been shown to promote the rewiring of glutamine in pancreatic ductal adenocarcinoma (PDAC) and CRC [49, 92, 93]. Recent evidence indicates that metabolic enzymes can be modulated using posttranslational changes, such as butyrylation, crotonylation, propionylation, glutarylation, methylation, acetylation, succinylation, and malonylation [94, 95]. Sirtuin5 (SIRT5), a member of the sirtuin family and a regulator of posttranslational modifications (PTMs), globally regulates lysine succinylation [96, 97, 98], malonylation [96, 99], and glutarylation [100]. However, the reduction of biological ROS is brought about by the desuccinylation of IDH2 and the deglutarylation of glucose‐6‐phosphate dehydrogenase by SIRT5, which shields cells from oxidative damage [101]. In CRC tissues and cells, SIRT5 is highly elevated [102]. It sustains the TCA cycle and enhances glutaminolysis by activating GLUD1 through deglutarylation, making SIRT5 a potential target for anti‐CRC therapy. To assist cancer cells overcome oxidative stress barriers during carcinogenesis, mutations in KEAP1 trigger the nuclear factor erythroid‐related factor 2 (NRF2) antioxidant program and work in tandem with mutant KRAS to drive the development of lung adenocarcinoma (LUAD) [103, 104, 105, 106, 107, 108]. Other malignancies with genetic mutations may be open to therapy due to the metabolic need for glutaminolysis in KEAP1–NRF2‐mutant LUAD tumors [109, 110, 111, 112] like epigenetic [113, 114], or posttranscriptional changes [115] in the KEAP1–NRF2 axis.

Furthermore, ammonia release from tumor cells involved in glutaminolysis acts as an autocrine and paracrine signal, promoting autophagy and protecting cells in different tumor regions from internal or environmental stress [20]. Recent findings in BC patients indicate an inverse correlation between GLS2 levels and epithelial–mesenchymal transition (EMT). Decreased GLS2 expression is associated with reduced mitochondrial activity. FOXC2, a critical regulator of EMT, plays a role in this process. Inhibiting FOXC2 expression restores GLS2 expression and enables glutamine utilization. These findings suggest that epithelial cancer cells undergoing EMT become independent of glutamine. Inhibiting EMT induces a metabolic shift directed by GLS2 in mesenchymal cancer cells, making them more susceptible to chemotherapy. Further validation is required to explore the predictive value of the inverse relationship between GLS2 and FOXC2 in BC outcomes [116].

## 4 Signaling Pathways Regulating Glutamine Metabolism

Glutamine metabolism is regulated by various signaling pathways crucial for disease and cellular homeostasis. Several critical signaling pathways, including c‐Myc, mTOR, and KRAS, are tightly linked to the regulation of glutamine metabolism. The mTOR pathway, a key regulator of cell growth and metabolism, enhances glutamate influx and metabolism in cancer cells, making it a potential therapeutic target. Other signaling pathways, such as AMPK, AKT, and Wnt/β‐catenin, have been implicated in regulating glutamine metabolism and offer potential avenues for therapeutic intervention. Understanding the intricate signaling networks that control glutamine metabolism opens up opportunities for developing novel therapies for diseases associated with dysregulated glutamine metabolism, including cancer, neurodegenerative disorders, and metabolic syndromes (MetS). Further research into these signaling pathways and their specific roles in glutamine metabolism will be crucial for developing effective targeted therapies and identifying diagnostic biomarkers for various diseases.

### 4.1 KRAS Signaling Pathway‐Mediated Glutaminolysis

Numerous cellular oncogenes and associated pathways in cancer cells regulate GLS1 activity [117]. One of these genes is KRAS, which is frequently altered in malignancies and enhances the growth of cancer cells. Cancer cells transformed by KRAS exhibit a high dependence on glutamine for growth proliferation [118] by increasing the production of AKG [119]. Approximately 20% of KRAS‐mutant LUAD tumors possess loss‐of‐function mutations in the KEAP1 gene, which encodes Kelch‐like ECH‐associated protein 1 [120, 121]. Moreover, more than 90% of persons with PDAC exhibit KRAS mutations and heavily rely on glutamine for survival [122]. Recent studies have shown that in PDAC cells, mutant KRAS transcriptionally activates NRF2, a key regulator of cellular redox, leading to a reduced intracellular environment [107, 123, 124], which increases the dependency on glutamine.

Furthermore, the expression of mutant KRAS enhances the production of the amino acid antiporter SLC7A5, which transfers glutamine in return for other amino acids to satisfy cellular requirements. This process promotes the growth of tumor cells in vitro and in vivo [125]. NRF2 significantly increases intracellular glutamate utilization by stimulating glutamate discharge, GSH production, and cystine consumption. Additionally, KEAP1 alterations enable cancer cells to survive oxidative stress by activating the NRF2 antioxidant machinery and collaborating with altered KRAS to promote LUAD development [103, 104, 105, 106, 107, 108]. The KRAS mutation condition and the metabolic rewiring features related to NRF2 addiction provide potential insights for innovative treatment approaches to target NRF2‐addicted tumor cells. Targeting KRAS directly or its downstream effectors has proven ineffective, highlighting the need for new therapeutic strategies for KRAS‐mutant CRC [126, 127]. KRAS, a significant oncogene in CRC, rewires glutamine metabolism and promotes succinate production from α‐KG. Using isogenic cell lines expressing wild‐type or mutant KRAS revealed that mutant KRAS decreases glucose reliance in colon cells, favoring glutamine utilization [128]. Genetically T cells that had been engineered to clonally express two allogeneic HLA‐C*08:02‐restricted T‐cell receptors (TCRs) targeting mutant KRASG12D expressed by the tumors [129]. In addition, strategies such as using LODER‐driven si^G12D^ to inhibit KRAS expression or covalent inhibitors of KRAS^G12C^, such as ARS‐853, have demonstrated inhibition of mutant Kras‐driven signaling and tumor growth in preclinical models [130].

While the usual course of treatment for individuals with KRAS wild‐type colon cancers (CC) is anti‐EGFR therapy, patients with RAS mutant tumors are excluded from this treatment [131]. It has also been discovered that more than two‐thirds of the proteome associated with oncogenic KRAS is regulated nonautonomously by factors secreted by activated fibroblasts. Chemical probes such as BI‐2852 and BAY‐293, which inhibit pan‐KRAS, have shown effective antitumor potency in PDAC, preventing the growth of cells in three‐dimensional organoids grown from patient tissues [132]. Targeting KRAS mutations is attractive because of their high occurrence and significance in tumor development. New approaches, including NMR‐based fragment screening, tethering, and in silico drug design, have been employed to identify compounds that bind directly to KRAS [133]. However, further research is needed to assess clinical safety, improve effectiveness, and overcome medication resistance. Additional research is required to find effective therapy alternatives.

### 4.2 Myc Signaling Pathway and Glutamine Metabolism

Myc is a pleiotropic transcription factor that regulates cellular processes such as proliferation, differentiation, metabolism, ribogenesis, and bone and vascular formation. Interestingly, Myc has been discovered to play an unanticipated role in different types of cancers. Myc‐induced glycolysis in vivo was proven by utilizing transgenic mice models in which Myc was overexpressed in hepatocytes, resulting in increased production of glycolytic enzymes and glycolysis [134]. Myc is crucial for cancer development and is frequently found in cancer cells that exhibit resistance to anticancer drugs [135]. MycL and MycN are members of the Myc family, which is crucial in controlling the reprogramming of metabolism in various human types of tumors [136]. Overexpression of Myc leads to apoptosis by converting prosurvival signals (such as bcl‐2) to prodeath signals (such as bid) [137]. c‐Myc controls the expression of several genes that play vital roles in glutamine metabolism, including GLS1, GLUD, and aminotransferases [24, 52, 138]. The de novo synthesis of glutamine from glutamate and ammonia is catalyzed by GLUL, whose expression is enhanced by Myc. The process involves the activation of thymine DNA glycosylase, Myc's transcriptional target, causing the Glutamine synthetase (GS) promoter to undergo active demethylation and exhibit higher expression. In Myc‐caused malignancies, these pathways imply a unique biological relationship between glutamine metabolism and DNA demethylation [24]. Furthermore, the proto‐oncogene c‐Myc is associated with glutamine metabolism by transcriptionally binding to the promoter regions of glutamine importers, including ASCT2 (sodium‐dependent neutral amino acid transporter type 2, also known as SLC1A5) and SN2 (an isoform of system N, also known as SLC38A5), resulting in increased glutamine uptake [50]. C‐Myc controls glutamine uptake and conversion via transporters such as SLC3A2, SLC7A1, SLC5A1, and GLS‐1 and GLS‐2 [50]. It promotes glutamate conversion into α‐KG by favorably modulating glutamate dehydrogenase (GDH), glutamic oxaloacetic transaminase (GOT), and ornithine‐delta‐aminotransferase. C‐Myc‐dependent glutamine catabolism supplies intermediate metabolites of the TCA cycle, creating α‐KG and promoting amino acid, nucleotide, and lipid synthesis [139]. This abundance powers the TCA cycle, triggering OXPHOS and activating the electron transport chain (ETC). Being overexpressed in many tumor cells, Myc is considered one of the most common and aggressive oncogenes. It is frequently linked to treatment resistance and a bad prognosis for cancer patients. The study provides insights into cancer resistance mechanisms and proposes possible c‐Myc and glutamine metabolism therapies. Targeting both may improve therapeutic outcomes, indicating a promising option for future cancer therapies.

### 4.3 Autophagy and mTOR Signaling Pathway‐Mediated Glutaminolysis

The mTOR pathway is important as a crucial signaling junction, as is widely known, and it is now thought that mTORC1 activation is linked to glutaminolysis [140]. It has been determined that glutamine is an essential nutrient for various tumor forms, especially when the tumor TME is nutrition deficient [7, 141]. The coinduction of glutaminolysis by glutamine and leucine activates the mTORC1 signaling pathway, which promotes cell growth by enhancing αKG production and inhibiting autophagy [17]. α‐KG, a glutamine metabolic byproduct, stimulates mTORC1 and promotes Rag‐mediated GTP loading [17]. Treatment with glutamate and leucine raises ATP levels, inhibiting AMPK and activating mTORC1. ASNS and GLS dual repression on mTORC1 in U1OS cells inhibit mTOR activity only when both are present [80]. This shows that ASNS operates as an alternate glutamine route, impacting metabolism and mTORC1 activation. The translocation of mTORC1 to the lysosome occurs when GTP binds to RagB in the RagB–RagC heterodimer complex. Lysosomal translocation and activation of mTORC1 are stimulated by increased glutaminolysis or an analog of cell α‐KG. Glutaminolytic α‐KG enhances the GTP loading of RRAG proteins (regulators of lysosomal signaling and trafficking), activating mTORC1 and inhibiting autophagy [142, 143]. Amino acids are crucial regulators of mTORC1, which promotes anabolic pathways and represses catabolic processes like macroautophagy under nutrient‐rich conditions [144]. mTORC1 controls autophagy through Unc‐51‐like kinase 1 (ULK1), an upstream autophagy‐related protein. ULK1 forms a complex with multiple proteins to initiate autophagy, and mTORC1 associates with this complex, phosphorylates, and inhibits ULK1 and ATG13 to repress autophagy [145]. This regulation prevents futile cycles of protein synthesis and catabolism.

On the other hand, blocking glutaminolysis stops RagB from binding GTP, which stops lysosomal trafficking and triggers the expression of mTORC1 [17]. Glutaminolysis results in the accumulation of excess ammonia within cells, and high ammonia levels can potentiate autophagy [20]. Autophagy is also enhanced by nutrient deprivation in the microenvironment, which activates FOXO3. FOXO3 facilitates the expression of GLUL, the enzyme that resynthesizes glutamine from glutamate [146]. This abrogates the production of αKG from glutaminolysis, inhibits mTORC1, and enhances autophagy [142, 147]. The ULK complex, which comprises ATG13, RB1CC1/FIP200, ATG101, and ULK1/2, becomes activated when mTORC1 is inhibited, which enhances autophagy [148]. EGLNs (prolyl hydroxylases), oxygen sensors in the cell, act as crucial regulators of αKG‐dependent activation of mTORC1 [148]. Numerous tumor suppressor genes, such as TSC1, TSC2, and phosphate and tensin homology (PTEN), promote autophagy and block upstream mTOR signaling. On the other hand, oncogene products that activate mTOR, such as class I PI3K and Akt, inhibit autophagy [149]. The connection between glutaminolysis, autophagy, and mTORC1 presents promising targets for developing therapeutic strategies against cancer. Inhibiting EGLNs, which link glutaminolysis to mTORC1 activation, could effectively inhibit mTORC1 activity. Furthermore, the inclusion of the Klotho protein can enhance the formation of the ULK1 complex transcription factor EB nuclear translocation and block the IGF‐1/PI3K/Akt/mTOR signaling pathway, all of which are essential for autophagy activation [150]. Natural and synthetic α, βα, β‐unsaturated carbonyls have shown anticancer properties by targeting mTOR, making them potential candidates for BC treatment. According to a study, A146Ply may serve as a novel autophagy suppressor for leukemia treatment. In K562 cells, the synergistic suppression of autophagy and activation of apoptosis was achieved with the combination of ΔA146Ply and CQ, a clinically accessible autophagy inhibitor [151]. AOS‐SO4 blocks the MEK1/ERK/mTOR signaling pathway, which is involved in various human malignant tumors and promotes angiogenesis and cell growth [152]. β‐Elemene and Puerarin are natural plant extracts derived from Rhizoma Zedoaria known for their anticancer properties against various types of cancers [153]. These extracts induce apoptosis through different mechanisms, including the PI3K/AKT/mTOR pathway [154, 155, 156]. Another natural product, Gypenosides, also exhibits potent anticancer effects by targeting the PI3/AKT/mTOR pathway. It achieves this by inhibiting the activity of Son of Sevenless, RAS, uPA, and FAK, inhibiting PI3K and Rho‐A, and regulating different pathways [157].

Glutaminolysis, which supplies energy and metabolic substrates, significantly influences autophagy. It activates mTORC1, thereby inhibiting autophagy, but recent studies also suggest that glutamine can regulate mTORC1 and autophagy independently of glutaminolysis. In this process, glutamine is converted through asparagine synthesis, and the gamma‐aminobutyric acid (GABA) shunts to generate ATP and inhibit AMPK. Prolonged glutaminolysis can maintain autophagy inhibition dependent on mTORC1, even without other amino acids. Interestingly, these studies also uncovered a link between excessive glutaminolysis without other amino acids and increased cell death [158]. Understanding the function of the mTOR signaling pathway in various biological processes and diseases has advanced significantly. However, to create innovative combinatorial medicines that modify autophagy pathways in cancer for the best possible therapeutic outcomes, it is imperative to comprehend the molecular mechanisms behind mTOR downstream activities, including autophagy.

## 5 Role of MicroRNAs in the Regulation of Glutaminolysis

MicroRNAs, small noncoding RNAs, regulate biological processes like gene expression and RNA silencing. Around 2200 conserved miRNAs have been identified, affecting cell growth, differentiation, metabolism, viral infection, and tumorigenesis [159]. MiRNAs are also involved in pathological settings, with cancer being a leading area of investigation. MiRNAs regulate energy metabolism in tumors, affecting genes and enzymes involved in metabolic pathways [160] (Table 1). Understanding miRNAs' role in these pathways is crucial for developing new therapeutics and identifying biomarkers for cancer diagnosis.

**TABLE 1:** miRNA‐guided reprogramming of glutamine metabolism in cancer.

S.No	miRNA	miRNA upregulated/downregulated	Direct target	Pathway	Cancer type	References
1	miR‐105	Upregulated	MXI1	Glutaminolysis	Breast cancer	[161]
2	miR‐9‐5p	Downregulated	GOT1	Glutaminolysis	Pancreatic cancer	[162]
3	miR‐18a	Downregulated	GCLC	Glutaminolysis	Liver cancer	[163]
4	miR‐145	Downregulated	c‐Myc	Glutaminolysis	Ovarian cancer	[164]
5	miR‐203	Downregulated	GLS	Glutaminolysis	Melanoma	[165]
6	miR‐153		GLS	Glutaminolysis	Glioblastoma	[166]
7	miR‐137	Downregulated	ASCT2, SLC1A5	Glutaminolysis	Glioblastoma, colorectal cancer, pancreatic ductal adenocarcinoma, prostate cancer	[167]
8	miR‐122	Downregulated	GLS	Glutaminolysis	HCC	[168]
9	miR‐1‐3p	Downregulated	GLS	Glutaminolysis	Bladder cancer	[169]
10	miR‐122	Downregulated	SLC1A5	Glutaminolysis	HCC	[168]
11	miR‐450a	Downregulated	ACO2, TIMMDC1, ATP5B, and MT‐ND2	Glutaminolysis	Ovarian cancer	[170]
12	miR‐140‐5p	Downregulated	Glutamine synthetase	Glutaminolysis	Glioma progression	[171]
13	miR‐133a‐3p	Downregulated	Gamma‐aminobutyric acid receptor‐associated protein‐like 1 (GABARAPL1) and ATG13	Glutaminolysis	GC	[172]
14	miR‐23a/b	Downregulated	GLS, *ATG12*	Glutaminolysis	Prostate cancer, HCC	[52, 173]
15	miR‐513c	Downregulated	GLS	Glutaminolysis	Neuroblastoma	[174]
16	miR‐103a‐3p	Upregulated	GLS2	Glutaminolysis	GC	[175]
17	miR‐1	Upregulated	OXPHOS	Glutaminolysis	Leukemia	[176]
18	miR‐141‐3p	Downregulated	GLS	Glutaminolysis	Osteosarcoma	[177]
19	miR‐3163	Upregulated	GLS	Glutaminolysis	BC	[178]

## 6 Role of lncRNAs in the Regulation of Glutaminolysis

Eukaryotes possess a diverse array of RNA molecules that are crucial for transmitting genetic information and often exhibit specific subcellular localization. RNA synthesis, processing, and transport play crucial and interconnected roles in controlling various cellular activities and functions. Depending on their size, ncRNAs can be categorized into many categories, such as transfer RNA‐derived short RNAs, PIWI‐interacting RNAs, miRNAs, small nucleolar RNAs, and newly identified category of lncRNAs. Over 68% of the genes expressed in the human transcriptome are transcribed into lncRNAs. LncRNAs are comparatively large RNA transcripts that span more than 200 nucleotides, possess minimal or no capacity to code for proteins and exhibit restricted conservation among different species [179]. Various forms of cancer frequently exhibit changes in lncRNAs, which impact metabolic reprogramming, a characteristic of cancer (Table 2). It is unclear exactly how lncRNAs control these biological processes. Still, they control glutamine uptake‐a vital fuel source for cancer cells, supporting their survival and growth [180].

**TABLE 2:** LncRNAs affecting the glutaminolysis pathway in cancer.

S. no	LncRNA	Target	Disease	References
1	TUG‐1	miR‐145	ICC	[183]
2	EPB41L4A‐AS1	HIF‐1α, ATF4, ROS	CC, BC, BDC, HCC	[184]
3	OP15‐AS1	miR‐127	Melanoma	[185]
4	P21	GLS	BDC	[186]
5	GLS‐AS	c‐Myc/GLS	PDAC	[187]
6	HOTTIP	miR‐129, miR‐204	HCC	[188]
7	UCA1	miR‐16	BDC	[189]
8	HOTAIR	miR126‐5P	GBM	[190]
9	CCAT2	GLSPre mRNA, CFIm25	CRC	[191]
10	lncRNA IDH1‐AS1	α‐KG, ROS	CLC, LC	[192, 193]

Abbreviations: BC, breast cancer; BDC, bladder cancer; CC, cervical cancer; CC, colon cancer; CRC, colorectal carcinoma; GBM, glioblastoma; HCC, hepatocellular carcinoma; ICC, Intrahepatic cholangiocarcinoma; LC, lung cancer; PDAC, pancreatic ductal adenocarcinoma; α‐KG, α‐ketoglutarate.

**FIGURE 2:**
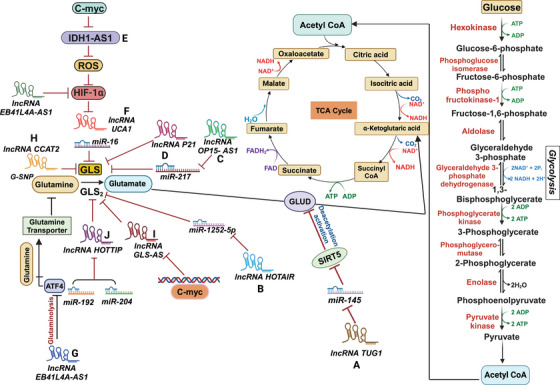
LncRNAs regulate glutamine metabolism in cancer. (A) In intrahepatic cholangiocarcinoma. IncRNA TUG1 has been shown to increase glutamine metabolism by inhibiting miR‐145and thereby preventing the degradation of mitochondrial protein Sirtuin 3 (Sirt3) mRNA enables GDH to become deacetylated, which enhances its activity. (B) HOTAIR regulates GLS expression by acting as a competing endogenous RNA (ceRNA) for the miRNA‐126‐5p, thus altering the glutamine metabolism process of glioma and promoting the development of tumor growth. (C) OIPS‐AS1 functions as a miR‐217 sponge to increase the expression of GLS, thereby upregulating glutaminolysis, contributing to the malignant development of melanoma tumors. (D) (lincRNA‐p21), which downregulated in cancer and increase glutamine metabolism and increase expression of GLS even though the mechanisms are still unknown. (E) In prostate cancer. IncRNA IDH1 antisense RNA1 (IDH1‐AS1) enhances IDH1 enzymatic activity at the posttranslational level by facilitating IDH1 homodimerization, resulting in increased production of α‐ketoglutarate (α‐KG). (F) UCA1 acts as a sponge for miR‐16, impairing canonical miR‐16 binding to the 3'UTR of GLS2 mRNA and preventing GLS2 mRNA degradation and is transcriptionally upregulated by HIF‐1a under hypoxia. thereby contributing glutamine metabolism in bladder cancer. (G) EPB41L4A‐AS1 eventually inhibits glycolysis and glutaminolysis in cancer cells by reducing HIF‐1α protein levels and inactivated downstream p‐elF20/ATF4 pathway but Low expression of the IncRNA EPB41L4A‐AS1, is involved in glutaminolysis. (H) Overexpression of CCAT2 predominantly G allele in the CC cells increases intracellular and extracellular glutamate, which correlates with the high activity of GLS. (I) In pancreatic cancer, IncRNA GLS‐AS expression is downregulated and participates in cancer metabolism, which is regulated by the c‐Myc transcriptional factor, thereby increasing the expression of GLS2. Reciprocal feedback, where GLS‐AS overexpression inhibited GLS expression and decreased c‐Myc protein levels in a proteasome‐dependent way. (J) HOTTIP overexpression raises GLS1 expression levels and enhances glutamine metabolism, and there occurs a suppression of IncRNA HOTTIP induced by miR‐192. miR‐204.

Interestingly, lncRNAs and other factors can regulate and activate different isoforms of GLS in distinct ways, and their upregulation is linked to a higher chance of developing certain malignancies [181, 182]. Therefore, the internalization and metabolism of glutamine are critical for multiple biological processes in cancer cells, including energy production, synthesis of macromolecules, and the maintenance of redox equilibrium and levels of ROS [11]. Consequently, glutamine is involved in many biological functions, and lncRNAs target various enzymes involved in glutamine metabolism (Figure 2).

### 6.1 LncRNA TUG1

In the context of ICC, an aggressive type of liver cancer, It has been shown that taurine upregulates gene 1 (TUG1), a lncRNA, contributes to increased glutamine metabolism via suppressing miR‐145. Upregulation of lncRNA TUG1 in ICC has been associated with poor prognosis and unfavorable clinical and pathological outcomes [183]. TUG1 acts as a sponge for miR‐145, preventing its normal function. miR‐145, when not inhibited by TUG1, targets and suppresses the expression of Sirtuin 3 (Sirt3) mRNA (Figure 2A). The protein Sirt3 in the mitochondrial matrix is involved in the deacetylation and activation of GDH [194]. By deacetylating GDH, Sirt3 enhances its activity, promoting glutamine consumption, α‐KG synthesis, ATP production, and positively regulating GDH translation [195]. In ICC cell lines, the knockdown of TUG1 leads to the suppression of Sirt3 mRNA by miR‐145, resulting in the hyperactivation of GDH. This hyperactivation leads to increased glutamine metabolism and related cellular processes [194].

### 6.2 LncRNA EPB41L4A‐AS1

It has been demonstrated that lncRNA can regulate the transcription of critical intermediates involved in glutamine metabolism through epigenetic modifications. EPB41L4A‐AS1 is one such lncRNA linked to glycolysis and glutaminolysis. A low level of EPB41L4A‐AS1 expression is associated with poor clinical outcomes [184]. EPB41L4A‐AS1 prevents the nucleoplasm translocation of HDAC2, a histone deacetylase, and its subsequent occupation of the VHL and VDAC1 gene promoters. EPB41L4A‐AS1 improves the connection between HDAC2 and NPM1 in the nucleolus, enhancing histone acetylation and transcription of VHL and VDAC1 genes. This, in turn, results in reduced levels of the HIF‐1α protein and inhibition of the downstream p‐eIF2/ATF4 pathway. HIF‐1α induces the transcription of genes involved in glycolysis, whereas ATF4 stimulates the development of amino acid transporters. EPB41L4A‐AS1, therefore, performs a regulatory role in cellular metabolism by inhibiting glutaminolysis and glycolysis in cancerous cells (Figure 2G) [184].

Moreover, peroxisome proliferator‐activated receptor gamma coactivator 1‐α and p53 can induce the expression of EPB41L4A‐AS1. Its low expression and loss are associated with poor prognostics in various human malignancies. Elevated glutamine and aerobic glycolysis metabolism result from EPB41L4A‐AS1 loss, and as a result, intercellular glutamate and α‐KG levels drop. Additionally, cancer cells become more dependent on glutamine when EPB41L4A‐AS1 is deleted. It is interesting to note that elevated ROS causes ATF4 to activate. This, in turn, causes the SNAT5 (SN2) transporter to become overproduced, which raises glutamine utilization [184].

### 6.3 LncRNA OIP5‐AS1

It has been found that melanoma tumors have a considerable upregulation of the lncRNA opa‐interacting protein‐5 antisense transcript 1 (OIP5‐AS1). In patients with melanoma, an elevated level of OIP5‐AS1 is an independent risk factor for decreased survival [185]. OIP5‐AS1 knockdown reduces glutamine intake and cell proliferation in melanoma cells A375 and SK‐MEL‐1. Furthermore, the production of ATP and levels of glutamate and α‐KG are also suppressed. It may be inferred from this that OIP5‐AS1 may be involved in the growth and progression of melanoma cancers by elevating glutaminolysis, a mechanism that breaks down glutamine [185]. OIP5‐AS1 is hypothesized to serve as a “sponge” for the microRNA miR‐217, which explicitly targets GLS. OIP5‐AS1 functions as a miR‐217 sponge, promoting the upregulation of GLS, a gene involved in glutamine metabolism. This finding suggests a potential mechanism through which OIP5‐AS1 regulates glutaminolysis and influences melanoma tumor progression(Figure 2C) [185].

### 6.4 LincRNA‐p21

LincRNA‐p21 is a type of lncRNA that is downregulated in cancer. It has been discovered that lincRNA‐p21 can regulate glutamine catabolism, a process involved in the breakdown of glutamine. In studies carried out on bladder tumor cells, exogenous production of lincRNA‐p21 was found to inhibit cellular growth and proliferation. Conversely, when lincRNA‐p21 was silenced, the opposite effect was observed. Additionally, overexpression of lincRNA‐p21 decreased GLS transcripts and proteins within the cells.

Consequently, the levels of glutamate and α‐KG also declined. The glutamine catabolism could be restored when GLS was overexpressed in lincRNA‐p21 knockdown cells. The findings suggest that lincRNA‐p21, which relies on GLS activity, may suppress the tumor by controlling glutamine catabolism Figure 2D). However, the precise mechanisms underlying this regulation are still unknown [186].

### 6.5 LncRNA GLS‐AS

The role of glutaminase's nuclear‐enriched antisense lncRNA (GLS‐AS) in PDAC metabolism has been discovered. It was found that individuals with PDAC who had downregulated GLS‐AS expression had a worse overall survival rate. In vitro and in vivo silencing of GLS‐AS causes an upsurge in cell invasion and multiplication in PDAC cells. Interestingly, GLS‐AS interferes with the posttranscriptional expression of GLS through an ADAR/dicer‐dependent RNA interference (RNAi) mechanism. GLS‐AS was downregulated, while GLS mRNA and protein were upregulated in response to glutamine and glucose deprivation. This indicates that the possible dysregulation of GLS‐AS and GLS may react to nutritional deprivation stress.

Additionally, the study showed that c‐Myc attaches to the GLS‐AS promoter and suppresses its transcription, a process made worse by malnutrition. However, when c‐Myc was knocked down, GLS‐AS expression increased after glucose and glutamine deprivation. There was evidence of a reciprocal feedback loop in which overexpression of GLS‐AS reduced c‐Myc protein abundance in a proteasome‐dependent fashion and suppressed GLS expressions (Figure 2I).

On the other hand, GLS upregulation maintained c‐Myc after dietary stress. Ultimately, exogenous GLS‐AS expression impaired the c‐Myc/GLS pathway, decreasing PDAC cell invasion and multiplication. The results indicate that GLS‐AS interacts with the c‐Myc pathway and modulates GLS production to influence PDAC metabolism. Treatment for PDAC may benefit from modulating the GLS‐AS/GLS/c‐Myc axis [187].

### 6.6 LncRNA HOTTIP

The study focused on the lncRNA HOTTIP (HCC's oncogene) role in HCC and its involvement in GLS1‐mediated glutamine metabolism. The findings indicated that overexpression of HOTTIP could increase the expression levels of GLS1 and enhance glutamine metabolism in HCC. Further analysis in HCC cell types demonstrated that miR‐192 and miR‐204 caused the transcriptional level reduction of HOTTIP via the argonaute 2‐mediated RNAi pathway (Figure 2J). Silencing HOTTIP using miR‐192 and miR‐204 led to a decrease in cell viability, suggesting the potential tumor‐promoting role of HOTTIP. On the other hand, cell proliferation was enhanced when HOTTIP breakdown was prevented by blocking the activity of miR‐192 and miR‐204. The research revealed that the HCC model's glutaminolysis might be disrupted by the miR‐192/‐204–HOTTIP axis, suggesting GLS1 as a putative downstream target. Intriguingly, HCC samples showed upregulated HOTTIP expression and downregulated miR‐192 and miR‐204 levels, suggesting a definite inverse relationship.

Furthermore, in HCC patients, alteration of the three ncRNAs (HOTTIP, miR‐192, and miR‐204) was linked to low life expectancy rates. In summary, the study highlighted the involvement of HOTTIP in HCC's glutamine metabolism through its regulation by miR‐192 and miR‐204. The dysregulation of this regulatory axis and its impact on glutaminolysis may have implications for HCC progression and patient prognosis [188].

### 6.7 LncRNA IDH1‐AS1

Several malignancies have elevated levels of cMyc, another carcinogenic transcription factor. The genes LDHA, GLUT1, HK2, PFKM, and ENO are among those it targets either directly or indirectly [196]. LncRNA prostate cancer (PCa) gene expression marker 1 is specifically expressed in PCa and acts as a coactivator of c‐Myc [34] and c‐Myc transcriptionally represses IDH1‐AS1 [192]. Under normoxia, it is studied that c‐Myc works with HIF1α to activate the Warburg effect by controlling a lncRNA, IDH1‐AS1. When IDH1‐AS1 is expressed, it encourages IDH1 to homodimerize, which increases IDH1's catalytic activity (Figure 2E). This led to a reduction in ROS generation, an increase in α‐KG, and the consequent decrease in the expression of HIF1α, which curtailed glycolysis [192, 193]. Therefore, HIF1α activates the Warburg effect when c‐Myc suppression of IDH1‐AS1 is present. However, other stimulants, such as the TCA cycle intermediates αKG, succinate, fumarate, and malate, may also control the hydroxylation of HIF‐1α in the absence of oxygen. [197]. The intricate networks of metabolic control, such as IDH1‐AS1 overexpression inhibiting cell proliferation, are demonstrated by the cMyc–(IDH1‐AS1)–IDH1–αKG/ROS–HIF1α axis that connects two of the most significant cancer metabolism effectors. On the other hand, IDH1‐AS1 silencing aided in the growth of cancer xenografts and cell division. Therefore, restoring IDH1‐AS1 synthesis could be a viable metabolic strategy for treating cancer [198].

### 6.8 LncRNA UCA1

The metabolic rewiring of tumors in the bladder has been linked to the lncRNA urothelial carcinoma‐associated 1 (UCA1). Increased levels of GLS mRNA and protein are linked to UCA1 overexpression, and these factors help BDC cells reduce ROS and boost mitochondrial glutaminolysis. This molecular mechanism can be attributed to the sponge‐like activity of UCA1, as it sequesters miR‐16 and blocks it from attaching to the GLS2 mRNA's 3'UTR region, thereby inhibiting the degradation of GLS2 mRNA (Figure 2F) [189]. Prior studies have demonstrated that UCA1 suppresses c‐Myc expression as a cancer suppressor in esophageal squamous cell carcinoma (ESCC) [199]. In bladder cancer, UCA1 plays a role in glutamine metabolism and blocks ROS generation by serving as a sponge for miR‐16 and increasing the levels of miR‐16, which targets GLS2. These data imply that UCA1 could be involved in controlling glutamine metabolism through multiple routes.

In summary, UCA1 is involved in the metabolic reprogramming of BDC by regulating glutamine metabolism. Its upregulation leads to increased GLS expression and subsequent modulation of ROS levels. UCA1 exerts this effect by serving as a miR‐16 sponge, influencing the levels of GLS2. These studies highlight the multifaceted role of UCA1 in regulating glutamine metabolism in bladder cancer [189].

### 6.9 LncRNA HOTAIR

HOTAIR lncRNA has been found to increase dramatically in glioma cells. Research studies have revealed that HOTAIR functions as a “sponge” for miR‐126‐5p, enhancing glutamine metabolism in gliomas. HOTAIR is a competitive internal RNA (ceRNA), sequestering miR‐126‐5p and regulating GLS expression (Figure 2B) [190]. MiR‐126‐5p has been reported to possess inhibitory effects in gastric and lung cancers (LC). Regarding gliomas, it explicitly targets GLS, which results in a notable decrease in GLS levels at both the mRNA and protein levels. Through the miR‐126/GLS pathway, the lncRNA HOTAIR modulates GLS expression, ultimately impacting the glutamine metabolism process in gliomas and promoting tumor growth. In conclusion, by “sponging” miR‐126‐5p, the lncRNA HOTAIR functions as a ceRNA, causing glioma cells to change their glutamine metabolism and upregulate the expression of GLS. This regulatory mechanism contributes to the progression of glioma and tumor growth [200, 201].

### 6.10 LncRNA CCAT2

The lncRNA CC‐associated transcript 2 (CCAT2) regulates glutaminolysis. In CC cells, overexpression of CCAT2 leads to increased intracellular and extracellular levels of glutamate, correlated with elevated GLS. According to prior studies, CCAT2 is found in the 8q24 location, home to the rs6983267 single nucleotide polymorphism (SNP) linked to cancer risk factors. The two variants of this SNP, G, and T alleles, have been associated with different risks of developing CRC, with a more significant risk associated with the G allele. Interestingly, both G and T alleles of CCAT2 resulted in higher levels of secreted glutamate in HCT116 cells overexpressing CCAT2. In contrast, only the G allele exhibited increased intracellular glutamate production and higher GLS activity (Figure 2H).

Furthermore, compared with the KGA isoform, the G allele of CCAT2 generated more excellent production of the GLS isoform GAC at the mRNA and protein levels. Although both isoforms share the same active site, GAC isoforms demonstrate higher catalytic activity and are more proficient in inducing TCA cycle intermediates. These results imply that alternative splicing favoring the GAC isoform of GLS is encouraged by the G allele of CCAT2. Subsequent analysis showed that the T allele of CCAT2 interacts with the CFIm68 subunit, whereas the G allele binds to the cleavage factor I (CFIm) complex, namely with the CFIm25 subunit. This interaction shows the fact that CCAT2 binds to the CFIm complex. Furthermore, the G variant of CCAT2 interacts with the GLS pre‐mRNA in intron 14, particularly with UGUA nucleotide sequences, which facilitates GLS alternative splicing and favors the production of the GAC variant. Moreover, in a xenograft mice model, the GAC isoform induced increased invasion and metastasis in CRC. These findings suggest that CCAT2, particularly the G allele, modulates glutaminolysis through alternative splicing of the GLS isoforms and is associated with enhanced tumor aggressiveness in CRC [191].

## 7 Circular RNAs‐Mediated Glutaminolysis in Cancers

CircRNAs are endogenous biomolecules with closed‐loop structures specific to cells and tissue and are resistant to exonuclease digestion [202, 203]. They can function as transcription regulators and microRNA sponges and have been linked to the progression of many human diseases, including cancer [204]. circRNAs have emerged as novel noncoding RNAs that play essential roles in various tumors [205], particularly in metabolic reprogramming [206, 207]. Several functional circRNAs associated with cancer have been identified [206, 207], including circ‐002013 [208], circ‐ABCB10 [209], circ‐0032821 [210], and has‐circ‐0006168, which are implicated in LC, BC, Gastric cancer (GC), and esophageal cancer, respectively. The circRNA circ‐HECTD1 is highly expressed in GC and promotes glutaminolysis by modulating the miR‐1256/USP5 axis. Increased expression of circ‐HECTD1 in GC is associated with overall survival. miR‐137, a tumor suppressor in several malignancies, including stomach cancer, is another possible target of circ‐HECTD1 [211, 212]. Depletion of circHECTD1 enhances sensitivity to drug treatment via the miR‐137/PBX3 axis [213]. CircRNA circ‐0000517 interacts with miR‐330‐5p in NSCLC to enhance Yin yang‐1 (YY1) expression and boost cell proliferation and glutamine catabolism [214]. Previous research demonstrated that circRNA circ_0000003 facilitated GLS expression in tongue squamous cell carcinoma cells, indicating that circRNAs can regulate GLS expression and affect glutamine metabolism and cancer progression. According to several studies, miR‐330‐3p suppresses tumor growth in various tumor types and is inhibited by circ‐0016068, which causes PCa cells to proliferate, invade, and migrate more freely [215]. GLS was shown to be a putative target gene of miR‐579‐3p by target prediction and screening; however, more research is required to determine its precise function and mode of action in ESCC [216]. Furthermore, it was shown that circ‐0001093 was upregulated in ESCC tissues and cell lines and that circ_0001093 expression was positively connected with ESCC malignant phenotype and poor survival. It was discovered that circ‐0001093 may bind to the tumor repressor miR, miR‐579‐3p, and negatively regulate it [217]. According to a study, circ‐0001093 functions as a molecular sponge for miR‐579‐3p to promote GLS expression, glutamine metabolism, and the malignant phenotype of ESCC [218]. Similarly, the host gene of circ‐OGDH regulates the interaction between glutamine metabolism and the TCA cycle. Research findings showed that circ‐OGDH silencing reduced ATP content, α‐KG synthesis, glutamine consumption, and GLS1 protein level in ESCC cells, suggesting that circ‐OGDH promoted glutamine metabolism in these cells. Mechanical sponging inhibited miR‐615‐5p, allowing circ‐OGDH to release PDX1, enhancing glutamine metabolism, and supporting tumor growth in ESCC, suggesting circ‐OGDH as a promising therapeutic target [219].

Moreover, the control of autophagy has been linked to cirRNAs. Deletion of circRNAs enhances autophagy, leading to apoptosis and reduced proliferation in cervical cancer cells. For instance, circ‐cTICRR binds with HuR protein, which stabilizes GLUD1 mRNA and increases the level of GLUD1 protein, indicating its oncogenic role in cervical cancer. There may be hope for cervical cancer treatments if the circ‐TICRR relationship with the HuR protein is addressed [220]. Additionally, circ‐SLC25A16 induces a rise in the rate of extracellular acidification, ATP synthesis, intake of glucose, and lactate formation, all of which support A549 cell growth. It operates as a ceRNA by binding to miR‐488‐3p and enhancing the synthesis of HIF‐1α [221]. These studies highlight the multiple roles that circRNAs play in tumor development, including their involvement in metabolic reprogramming, modulation of miRNA activity, and regulation of vital cancer‐related genes. Targeting circRNAs and their associated pathways could have potential therapeutic implications for cancer treatment.

## 8 Diseases Associated with Glutaminolysis Deregulation

**FIGURE 3:**
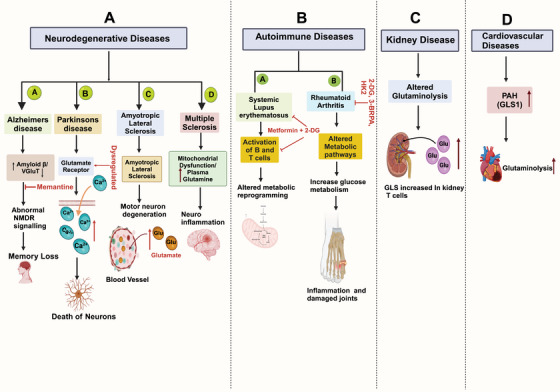
This figure illustrates the role of glutaminolysis in various diseases, including neurodegenerative disorders, autoimmune conditions, kidney diseases, and cardiovascular diseases. Key metabolic alterations in glutamate and glutamine pathways are highlighted, showcasing their impact on cellular function and disease progression. The potential therapeutic targets within these pathways are also emphasized, suggesting avenues for future research and treatment. Overall, glutaminolysis emerges as a critical factor influencing disease mechanisms across multiple biological systems.

Glutaminolysis, the metabolic pathway involving the conversion of glutamine to various intermediates, plays a crucial role in cellular function and energy production. This process has garnered significant attention due to its association with a range of diseases, particularly neurodegenerative diseases, Autoimmune diseases, kidney diseases, and cardiovascular diseases. Understanding the intricacies of glutaminolysis sheds light on the underlying mechanisms of these diseases and opens avenues for potential therapeutic interventions to target this metabolic pathway. Here, we discuss several diseases associated with glutaminolysis (Figure 3).

### 8.1 Neurodegenerative Disorders

A characteristic of neurodegenerative disorders is the degradation of neuronal clusters [222]. Numerous cellular and molecular abnormalities, such as glutamate toxicity, mitochondrial dysfunction, and neuronal death, are expected to cause neurodegenerative disorders [223]. These delicate neurons are more metabolically demanding to maintain their structural complexity, which renders them preferentially sensitive. They also contain complicated morphological traits, such as numerous synaptic connections [224, 225]. Numerous studies have examined how metabolism functions in intricate neurological disorders that strongly correlate to mitochondrial malfunction [226, 227, 228].

### 8.2 Alzheimer's Disease

Alzheimer's Disease (AD) is a progressive age‐related neurodegenerative illness that causes significant memory loss and cognitive impairment at the same time when amyloid plaque builds up in neocortical and hippocampus tissue [229]. AD results in abnormalities in the pulmonary and circulatory systems, reducing the amount of oxygen reaching the brain, nutritional deficiencies, vitamin B12 deficiency, tumors, and other things can all lead to a progressive loss of cognitive functions [230]. AD has a complicated, multifactorial origin. Genetic mutations in presenilin (PS1, PS2) and amyloid precursor protein (APP) genes cause early‐onset familial AD by disrupting a shared pathogenic pathway in APP synthesis and producing excessive amounts of amyloid β (Aβ) [231]. One key element influencing glutamate availability for signaling events is the absorption and recycling system. Undesirably, this mechanism may be seriously compromised in AD. In Alzheimer's disease patients, the expression and capacity of the vesicular glutamate transporter (VGluT) are diminished [232]. Reduced glutamate and increased glutamine have been observed in cortical tissue of AD patients [233]. Glutamate sensing‐based signaling neurons require N‐methyl‐d‐aspartate receptor (NMDAR), a glutamate‐responsive receptor, to function. However, when overstimulated, the receptor also plays a critical role in Ca^2+^ influx‐mediated excitotoxicity. Consequently, neurons may suffer catastrophic consequences from insufficient and excessive NMDAR signaling [234, 235]. Amyloid accumulation appears to decrease the expression of EAAT2 on astrocytes, impairing glutamate reuptake via the glutamate‐glutamine cycle and contributing to NMDAR‐mediated excitotoxicity [236]. Memantine, one of the few pharmacological treatments for AD, is an United States Food and Drug Administration (US FDA)‐approved moderate affinity antagonist for the NMDAR that was created to obstruct glutamate excitotoxicity [237]. Sadly, results from early trials are inconsistent, suggesting that memantine may have a limited advantage [238].

### 8.3 Parkinson's Disease

PD is a prevalent neurological illness that results in the death of neurons in the substantia nigra, an area of the brain essential for dopamine synthesis [229, 239]. Tremors, bradykinesia, rigidity, and postural instability are among the movement symptoms of PD [240]. A PD patient subgroup has affected neurons that develop intracellular inclusions known as Lewy bodies [241]. Lewy bodies and basal ganglia neuronal loss (about 70% of dopaminergic neurons in the substantia nigra pars compacta (SNpc) are the primary pathogenic features of PD. The SNpc experiences a marked increase in microglia activation and astrocyte death with this neuronal loss [240]. Epigenetic alterations are thought to impact the regulation of the glutamate transporter gene in the etiology of PD [242]. Increased Ca^2+^ influx exacerbates ROS levels, damages mitochondria, and makes cells more vulnerable to death by over‐activating NMDARs.

Furthermore, excessive AMPAR and KAR activation causes Na^+^ overload, which raises intracellular permeability and causes acute cellular edema. Because dopaminergic neurons in the SNpc are particularly vulnerable to oxidative stress, the rise in free radicals is particularly significant for the pathophysiology of PD [243, 244]. To compensate for dopaminergic signaling abnormalities, levodopa, a dopamine precursor, and dopamine receptor agonists are used in the pharmacological therapy of PD symptoms [241, 245]. Glutamate may also contribute to neurodegeneration in PD, even though the disease is mostly linked to dopaminergic neurons. Research indicates that PD is associated with dysregulated glutamate receptor expression and function. In animal models, NMDAR antagonist administration decreases PD symptoms like rigidity and akinesia and improves levodopa efficacy [239, 246]. However, NMDAR antagonist clinical trials in PD patients have demonstrated modest benefit, and glutamate most likely just alters the course of the disease [239].

### 8.4 Amyotrophic Lateral Sclerosis

The primary characteristic of the deadly neurodegenerative disease known as ALS is the selective degeneration of both the brain's upper and lower motor neurons as well as those in the brain stem and spinal cord [247, 248]. Muscle weakness, paralysis, and ultimately death result from the degeneration of these motor neurons, mainly as a result of respiratory failure [249]. Increased glutamate has been discovered in the cerebral spinal fluid and blood of ALS patients. Increased glutamate but unchanged glutamine were found in postmortem ALS patient brain samples; however, patient MRS tests revealed increases in glutamate and glutamine [250, 251]. ALS patients and disease models have been shown to exhibit altered glutamate receptor expression and activity.

Furthermore, genome‐wide association studies have revealed associations between genes unique to glutamatergic neurons and the risk of developing ALS [252, 253]. Investigations using pharmacological and genetic methods to modify glutamate receptor function and glutamate signaling have only slightly improved the results of ALS in mice [254]. Long‐term riluzole administration has increased the glutamate‐glutamine cycle and glucose metabolism in the rat prefrontal cortex and hippocampus [255, 256]. Targeting glucose metabolism has the potential to decrease the incidence and course of ALS, even though metabolic treatments for the disease receive little attention. Chemicals that improve glucose absorption and metabolism through PPP and glycolysis may be advantageous because they lessen oxidative stress and increase ATP synthesis [257].

### 8.5 Multiple Sclerosis

Multiple sclerosis (MS) is a neuro‐inflammatory disease that affects the spinal cord and brain. Interestingly, MS cooccurs with the primary hereditary mitochondrial disease LHON; this combination of diseases is known as Harding's syndrome or LHON‐MS [258, 259]. Research on MS and primary mitochondrial disease is actively focused on identifying and targeting the mitochondrial pathways responsible for inflammation, with the idea that these two conditions are causally related [260]. The data supporting glutamine metabolism in MS are inconsistent, but it appears to have a similar function in AD and ALS. A study found that MS patients had raised plasma glutamine levels. Several studies have revealed that MRS increases glutamine and glutamate in MS brains, and this rise is the most significant indicator of the condition [261, 262]. The most changed metabolites in the MS brain, according to MRS and a review of MRS literature, are glutamine and glutamate. The direction of these changes varies depending on the severity of the disease and the area of the white matter being studied. The quality of the data (magnetic field strength, analytic techniques, etc.) has a significant impact on resolution accuracy [263]. Mouse experimental autoimmune encephalitis models of MS have demonstrated that glutamine antagonism attenuates disease and glutamate excitotoxicity contributes to disease progression; however, it is unclear how relevant these models are to MS [264].

### 8.6 Bipolar Disorder

A long‐term, progressive mental illness, bipolar disorder (BD) is marked by fluctuations in mood, including manic catastrophic effects on patients [265]. Patients with this ailment have a higher risk of drug usage, metabolic and endocrine diseases, vascular disease, and psychological and medical comorbidities [266]. There is still much to learn about BD's etiology and illness processes. Much research points to BD's etiology as largely dependent on mitochondrial dysfunction. Studies on postmortem brains have shown aberrant distribution, size, and shape of mitochondria in BD patients, along with a significant and widespread reduction in nuclear gene expression controlling OXPHOS [267, 268]. In numerous brain illnesses linked to glutamatergic anomalies, high levels of glutamate and glutamine have been demonstrated to be correlated with cognitive impairment [269]. Many anticonvulsant drugs are used in the treatment of epilepsy and bipolar illness, as they are highly comorbid conditions. There have been similarities in the underlying pathophysiology reported; however, opinions on whether anticonvulsant modes of action can help mood stabilization and seizure reduction are not entirely agreed upon [270]. Glutamate metabolism similarities could shed some light on this problem. For instance, enhanced glycolysis, shown to rise five times during a seizure compared with normal function, is correlated with increased glutamate levels during seizures [271]. 50–60% of individuals with epilepsy have psychological symptoms, 12% have BD symptoms, and almost half of these individuals go on to receive a BD diagnosis [265]. It is interesting to note that, considering that bipolar illness and epilepsy share anticonvulsant drugs, new research suggests that the ketogenic diet, an additional epilepsy treatment that affects glutamate metabolism, may help treat BD [272]. It has been shown that ketones, such as acetoacetate and beta‐hydroxybutyrate, function as alternative energy substrates in the brain and have neuroprotective properties against neurological disorders like epilepsy. Thirteen randomized‐controlled trials and more than a century of clinical use of the ketogenic diet have shown that ketosis is an effective treatment for reducing seizures in epilepsy [273].

### 8.7 Autoimmune Disorders

Metabolism plays a crucial role in immune regulation, with various metabolic pathways like glycolysis, pentose phosphate, fatty acid oxidation, glutaminolysis, Krebs cycle, and OXPHOS modulating innate and adaptive immune cells [274]. Metabolic aberrations and metabolite changes are linked to inflammatory immune cell phenotypes in autoimmune disorders like systemic lupus erythematosus (SLE), rheumatoid arthritis (RA), multiple sclerosis, and type‐1 diabetes [274]. Since lymphocyte destiny is regulated by metabolic adaptability, metabolic reprogramming may play a role in the etiology of autoimmune disorders.

#### 8.7.1 Systemic Lupus Erythematosus

Metabolic programs control immune cell fate and function, which is crucial in autoimmune diseases. Major metabolic pathways and studies conducted in preclinical models or patients regulate immune cell activation and differentiation in lupus. Amino acids, particularly glutamine, play a vital role in immune function. Glutamine is a key component in energy production and immune activation, with increased lymphocyte levels such as CD4+ T cells activated through receptors and CD28 [138]. MetS is a group of metabolic abnormalities, including hypertension, obesity, dyslipidemia, hyperglycemia, and insulin resistance, with SLE patients at higher risk. SLE is an autoimmune disease with lymphocyte imbalance. Follicular helper T (Tfh) cells in lupus‐prone mice display a specific SLC expression signature, including amino acid transporters like Slc7a5, Slc7a10, ASCT2, and LAT1/CD98. Understanding the functional link between solute transporters and immune cell metabolic programming could unlock novel regulatory circuits of immune activation. However, the lack of reagents like antibodies, inhibitors, and cell‐specific deletions for many SLE members remains a major hurdle [275]. A study found that glutamine metabolism levels were elevated in splenic and peripheral blood mononuclear cells of SLE patients. CB839 treatment for 8 weeks alleviated lupus‐like symptoms, improved B cell depletion, and reversed hyperactivated pathways in MRL/lpr mice. CB839 treatment improved B cell depletion, adjusted Th1/TH2, and TH17/Treg imbalance, inhibited GLS1, reduced Tfh cell numbers, and activated B cells in lupus mice [276]. Adequate amounts of glutamine are necessary for IL‐1 induction by macrophages upon LPS stimulation, and most enter the TCA cycle and hexosamine pathway, causing M2 macrophage polarization upon IL‐4 stimulation [277]. Glutamine metabolism also modulates the immune responses of T and B cells and is required to respond to antigen receptor stimulation. Amino acid transporters are essential for effector T cell differentiation and function. Glutaminolysis is essential for maintaining T cell activation and proliferation, and blocking glutamine with 6‐diazo‐5‐oxo‐l‐norleucine (DON) inhibits activation‐induced proliferation in vitro [138, 278]. Enzymes involved in glutaminolysis are elevated in CD4+ T cells from lupus‐prone TC mice, suggesting that DON treatment may be therapeutic for SLE T cells [279]. CD4+ T cells from SLE patients and lupus‐prone animals show increased mitochondrial metabolism and glycolysis, suggesting that SLE patients experience altered intrinsic metabolism reprogramming [279]. T cells use phosphatidylinositol 3‐kinase (PI3K) and Akt through CD28 costimulation to enhance their glucose absorption and glycolysis during immunological responses [280]. The SLE characteristics in lupus‐prone mice were improved by CG‐5 inhibition of glucose transporters through the suppression of Th1 and Th17 cell development, the induction of regulatory T cells, the reduction of germinal center B cell proliferation, and the production of autoantibodies.

#### 8.7.2 Rheumatoid Arthritis

An autoimmune illness that causes chronic inflammation and damage to joints and extra‐articular tissues is called RA [281]. The primary pathological characteristics of RA include pannus development, inflammatory cell infiltration, synovial hyperplasia, and erosion of bone and cartilage, which eventually result in progressive joint degeneration [282, 283]. Patients with RA have changed metabolites, and by stimulating immune cells and synovial fibroblasts, these altered metabolic pathways can worsen synovial inflammation [284]. Glutamine is highly elevated in the synovial fluid of RA patients, providing carbons to the TCA cycle through glutaminolysis [285]. Glutamine could serve as a potential biomarker for RA patients due to its role in regulating the proliferation of RA fibroblast‐like synoviocytes (RA‐FLS) and its involvement in the disease's development. Research has shown that the expression of glutaminase‐1 and glutamine consumption are elevated in RA‐FLS.

Additionally, ornithine, a precursor to glutamate linked to RA, may influence TNF‐α expression through its activity related to glutamate levels. A study found that FLS from RA patients are metabolically distinct and rely more on glutaminolysis for proliferation and survival [286]. The study also found that when glutamine levels were restricted or GLS1 was inhibited, the proliferation of RA‐FLS was suppressed, indicating that glutaminolysis is crucial for sustaining cell growth in RA. An experimental component showed that administering a GLS1 inhibitor to a mouse model of RA reduced inflammatory arthritis symptoms, indicating a potential therapeutic pathway targeting metabolic processes [286]. RA‐FLS may be addicted to glutamine, similar to cancer cells. Glutaminase 1 is upregulated in Th17 cells, which rely more on glutaminolysis. The glutamine antagonist DON reduced Th17 splenic cells and suppressed arthritis when combined with rapamycin in a mouse disease model [39]. SLC7A5, a crucial amino acid transporter, induces proinflammatory cytokine secretion in RA monocytes and macrophages through leucine influx [287]. SLC1A5 is a transporter that ensures glutamine uptake in T cells during activation, enabling naïve CD4+ T cells to fulfill their glutamine needs [288]. It couples TCR and CD28 signals to activate the mTORC1 pathway, allowing clonal expansion [289]. SLC1A5 expression is crucial for Th1 and Th17 cell lineage commitment, potentially regulating the pathogenic differentiation of short‐lived effector T cells in RA patients [290]. However, no information exists on how tissue microenvironments affect metabolically regulated aspects of T‐cell differentiation. Understanding differences in glutamine availability, energy substitution under glucose deprivation, and its interference with mTORC activation is essential for understanding T cell pathology in RA. Furthermore, RA patients' T cells have unique metabolic changes that favor PPP over glycolysis, allowing RA T cells to become invasive and proinflammatory [291]. According to several studies, glycolysis inhibitors reduce inflammation in animal models by suppressing the aggressive phenotype of RA‐FLS and immune cells. By lowering cytokine production, proliferation, and migration, inhibition of glycolysis with 2‐DG, 3‐bromopyruvate (3‐BrPA), or 3‐(3‐pyridinyl)−1‐(4‐pyridinyl)−2‐propen‐1‐one reduces the aggressive phenotypes of RA‐FLS [292, 293]. Consequently, in animal models, therapy with 2‐DG or 3‐BrPA reduced inflammatory arthritis [293, 294]. Thus, by modifying the pathogenic functions of RA‐FLS and immune cells, RA can be treated with a unique strategy that targets metabolic reprogramming.

### 8.8 Kidney Diseases

Changes in glutaminolysis have often been reported in various types of kidney cells [295, 296]. When acute kidney injury (AKI) occurs, tubular epithelial cells are impaired, which causes leukocytes to release inflammatory cytokines and chemotactic proteins [297]. The genetic condition known as polycystic kidney disease, characterized by many fluid‐filled kidney cysts, depends on glutamine metabolism to promote cellular development and multiplication [298]. Typically, the kidney uses very little glutamine; however, during the metabolic acidosis phase of AKI, the kidney uses a significant amount of glutamine, metabolizing around one‐third of the plasma glutamine [299]. A recent study discovered that GLS activity is elevated in kidney T cells during ischemia AKI, and glutamine blocking with its antagonist JHU083 may lessen renal damage [300]. According to a different study, glutamine promotes the production of heat shock proteins, which may act as a protective mechanism against cellular injury [301].

Additionally, an earlier study showed that when glutamine was given as a single dosage after sepsis began, the expression of mediators associated with high mobility group box‐1 decreased. The kidneys’ level of oxidative stress dropped as well [302]. In a rat model of experimental myoglobinuria, Kim et al. [303] found that intraperitoneal glutamine inhibited c‐Jun N‐terminal kinase phosphorylation of 14‐3‐3, thereby mitigating tubular cell death after acute kidney damage. Glutaminolysis is inhibited in antineutrophil cytoplasmic antibody‐associated vasculitis and nephrotic syndrome, according to transcriptional profiling of kidney biopsy samples from patients [304].

### 8.9 Cardiovascular Diseases

The primary cause of pulmonary arterial hypertension (PAH) is the increased migration and proliferation of vascular cells, which leads to the creation of lesions that clog the pulmonary blood arteries [305, 306]. The fibrosis and vasoconstriction that accompany this aberrant vascular remodeling response raise the pressure in the pulmonary arteries, which ultimately leads to early death. While it is well recognized that mitochondrial malfunction and metabolic reprogramming provide the cellular characteristics of PAH, glutamine metabolism's significance is now becoming apparent. It has been observed that early pathologic events in PAH stimulate the proliferation of pulmonary ECs and SMCs by inducing GLS1 through Yes‐associated protein 1 with a PDZ‐binding motif [307]. In the pulmonary arterioles of the monocrotaline (MCT)‐induced PAH rat model, GLS1 expression has been reported to rise, and glutamine measured in isolated pulmonary ECs decreases, suggesting greater glutaminolysis and anaplerotic flow through the Krebs cycle [307]. By stimulating collagen translation and stability through αKG‐mediated mTOR activation and proline hydroxylation, the elevated glutaminolysis in PAH also worsens fibrosis by causing an aggressive hyperproliferation and arterial stiffening. It has been observed that pharmacological reduction of GLS1 activity disrupts this cycle and reduces arterial remodeling in rats with MCT‐induced PAH [308, 309]. GLS1 expression was elevated in patients with human immunodeficiency virus‐mediated PAH lungs and in rhesus macaque monkeys with simian immunodeficiency virus‐associated PAH [41]. The enhanced proliferation and apoptosis‐resistant characteristics of PVCs associated with vascular remodeling in PAH have been attributed to metabolic imbalance [310]. The metabolic switch from OXPHOS to glycolysis may not produce enough ATP to support the excessive growth of PVCs. Although energy generation is less helpful for glycolysis per glucose molecule, some previous research suggested that the pulmonary arteries contain enough glucose to support the creation of ATP, which drives cellular proliferation [311]. Several treatment approaches can be used to stop glutaminolysis. GLS1 is a particularly appealing target in light of the introduction of multiple small‐molecule allosteric inhibitors [312]. In addition to going after GLS1, it is essential to think about Glu dehydrogenase or amino acid transaminase sending Glu to the Krebs cycle. By interfering with the anaplerotic use of glutamine in the Krebs cycle, pharmacological inhibition of Glu dehydrogenase by epigallocatechin gallate and R162 or amino acid transaminase by aminooxyacetate slows tumor growth and may be helpful in vascular hyperproliferative disorders [313].

## 9 Glutaminolysis: Target for Therapeutic Intervention in Cancers

**FIGURE 4:**
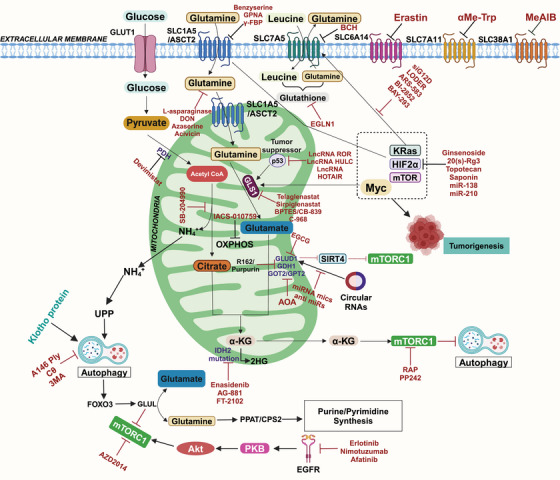
Targeting glutamine metabolic reprogramming in cancers. Glutamine and its involvement in glutaminolysis are critical in the metabolic network regulating cancer growth. To suppress cancer growth. strategies include using glutamine mimetics to target transporters and metabolic pathways like glutaminolysis, lipid synthesis, and autophagy. Enzymes involved in glutamine catabolism (GLS1, GLUD1. GDH1, and GOT2/GPT) and regulators of glutamine transport (KRAS. HIFO, mTOR) are targeted to inhibit glutamine breakdown and transport. Inhibiting glutamine synthesis from glutamine (by GLUL) reduces glutamine anabolism and nucleotide synthesis. EGFR and Akt/PI3K are targeted to inhibit mTOR. Tumor suppressor genes Myc and p53, which regulate glutamine metabolism and transport, are potential targets. miRNA mimics and anti‐miRs can inhibit circular RNAs that activate GLUD1. Glutamine‐driven oxidative phosphorylation, supporting ATP production, is inhibited by IASCS‐010759.

Various drugs are being tested in clinical trials to specifically target glutamine metabolism and inhibit glutaminolysis in different types of cancers (Figure 4 and Table 2). In addition, emerging therapeutic targets focus on mutated or overexpressed KRAS, which are present in multiple cancers. Clinical trials evaluate different inhibitors and targets of KRAS, with some inhibitors having already passed the initial clinical trial phase, while others are currently in phase 2 or 3 trials. One promising approach is targeting the downstream pathways of KRAS protein using MEK inhibitors such as selumetinib and trametinib. Selumetinib and docetaxel combination treatment significantly increased response rates for KRAS‐mutant NSCLC as compared with when docetaxel (NCT01933932) is used alone (NCT01933932) [314]. Phase 3 studies are still underway to assess the efficacy of docetaxel with a placebo or selumetinib in treating NSCLC with KRAS mutations [315, 316]. To successfully sequester KRAS in an inactive state, these efforts require finding allele‐specific drugs that disrupt the nucleotide exchange process and using a unique allosteric location in the binding pocket of the mutant cysteine residue [315, 316]. Clinical trials are also underway for EGLN1 inhibitors of KRAS, which are being tested for treating anemia [317]. These inhibitors have shown good tolerability and safety in early clinical trials.

Furthermore, the activation of AKT by the KRAS protein leads to the EphA2 phosphorylation at Ser897, resulting in increased cancer proliferation [318, 319, 320]. To target this pathway, there are ongoing phase 1 clinical trials for BT5528, a bicyclic peptide that binds to EphA2, and the phase 1 clinical trials for DS‐8895a, an EphA2 monoclonal antibody (Phase 1, NCT02252211), are complete. In many malignancies, KRAS and its downstream signaling cascades are the focus of several more recent clinical trials, such as  (NCT01274624, NCT03808558, NCT03785249, NCT04006301, NCT03600883, and NCT03948763) [321]. These trials aim to explore novel approaches and potential therapeutic options. Even though cancer cell‐targeted medicines have progressed, long‐term use can frequently result in relapse and resistance to drugs, highlighting the necessity for more study and development of more potent treatments.

The importance of autophagy in the development and progression of tumors with KRAS mutation may be one of the reasons for investigating its inhibition [322]. Targeting autophagy and other pathways, such as the ERK/MAPK pathway, is a strategy to improve outcomes in cancers with KRAS mutations. Clinical studies evaluate a range of medications targeting HIF‐1α, a protein involved in cancer growth. Some of these drugs include 2‐methoxyestradiol, which downregulates HIF‐1α at the posttranscriptional level [323], and tanespimycin, a heat‐shock protein 90 inhibitor that destabilizes HIF‐1α protein [324]. HIF‐1α protein expression is inhibited at the translational level by vorinostat [325]. Other drugs like EZN‐2968 and EZN‐2208 are being tested to inhibit the expression of HIF‐1α mRNA, leading to downregulating HIF‐1α protein in cancer cells [326, 327]. Autophagy, a cellular process, has gained attention in cancer research, particularly in Burkitt's lymphoma. The autophagy inhibitors chloroquine and hydroxychloroquine have been approved by the US FDA [328]. Through the neddylation path, the autophagy activator pevonedistat has shown potential as a novel inhibitor. Combination therapies targeting multiple therapeutic locations using autophagy inhibitors like chloroquine and pevonedistat have shown intriguing results in clinical trials [329]. Several additional autophagy blockers, including the Lys05 family [330, 331], ROC‐305 [332], and GNS561 [333], are currently being investigated in clinical trials. These inhibitors target the regulatory functions of lysosomes that are involved in autophagy. In addition to autophagy, lysosomotropic drugs like hydroxychloroquine have been found to inhibit micropinocytosis, which could potentially hinder tumor growth [334].

Moreover, these medications may have antitumor solid effects via lysosomal permeability independent of autophagy inhibition. Most evidence suggests that autophagy inhibition is beneficial in PDAC, and ongoing clinical trials explore the synergistic suppression of the ERK/MAPK pathway and autophagy. These trials focus on various ERK/MAPK pathway inhibitors combined with hydroxychloroquine. This combination therapy has shown promise in preclinical studies and is evaluated for its efficacy in clinical trials (NCT04145297, NCT03825289, NCT04132505) [322]. Inhibition of glutamine transporters to inhibit glutaminolysis in different cancers is also being investigated in NCT02771626 and NCT02071862 [335]. Clinical trials are underway for GLS inhibitors such as CB‐839 (NCT02071862, NCT02071888, and NCT02071927), as well as OXPHOS inhibiting molecules like atovaquone, metformin, IACS‐010759, and phenformin NCT03291938, NCT03026517, NCT01101438 and NCT03568994) [336, 337, 338]. MS0 (l‐methionine sulfoximine), a GS inhibitor, is additionally being explored as a possible treatment for glutamine‐addicted cancers [339, 340].

Many prospective paths are being investigated for cancer treatment, and targeting glutamine metabolism has emerged as a practical approach. Strategies include blocking receptors responsible for glutamine uptake, depleting glutamine levels in the plasma, and inhibiting glutamine synthesis and breakdown enzymes [341] (Table 3). In the case of acute lymphoblastic leukemia, targeting glutaminolysis has been demonstrated through the administration of l‐asparaginase, which reduces plasma glutamine concentration by converting glutamine to glutamate [342]. However, a limitation of using l‐asparaginase for targeting glutamine is the potential development of immunosuppression due to low glutamine levels required for the proliferation of immune cells [343]. Glutamine mimetics have been developed to inhibit glutaminolysis in various cancers. As an illustration, the glutamine analog DON acts as an antimetabolite and a GLS1 inhibitor. Another inhibitor, telaglenastat (CB‐839), explicitly targets GLS1 and has shown promising results in combination with paclitaxel, demonstrating tumor inhibition [344]. Telaglenastat has been proven to be effective in suppressing GLS1 in tumors in patients with multiple myeloma and lymphoma, either alone or in combination with other treatments [345]. MLN4924, a small molecule inhibitor, inhibits neddylation, a form of posttranslational modification responsible for controlling glutamine metabolism. This inhibits the degradation of the glutamine transporter ASCT2, which increases glutamine influx and utilization and enhances anticancer efficacy [346]. Blocking has been targeted at several plasma membrane glutamine transporters, including SLC7A11, SLC6A14, and SLC38A1. Agents like erastin, α‐Me‐Trp, and MeAIB have been found to inhibit these transporters, leading to altered glutamine metabolism [347]. Pharmacological targeting of the ASCT2 receptor using ‐l‐glutamyl‐p‐nitroanilide (GPNA) resulted in glutamine deprivation, specifically in ASCT2 overexpressing cells [348]. Another competitive antagonist of the transmembrane glutamine transporter, V‐9302, has shown promising results by attenuating cell growth, promoting cell death, and inducing oxidative stress, leading to antitumor responses [349]. Glutamine metabolism in cancer cells is a growing area of interest due to its role in various cellular functions beyond its metabolic role. Understanding how cancer cells coordinate these functions and reprogram their metabolic pathways in response to environmental stress could provide therapeutic intervention opportunities. However, challenges remain, and more specific pharmacological interventions targeting cancer cells' vulnerabilities will be the future focus of the field.

**TABLE 3:** Small molecule inhibitors targeting glutaminolysis pathway.

Approved cancer metabolic inhibitors/drugs					
S. no.	Small molecule inhibitors	Target enzyme	Mode of action	Cancer type	References
1	Enasidenib	Mutant IDH2	2‐Hydroxyglutarate synthesis	AML	[353]
2	Ivosidenib, LY3410738, DS‐1001b, IDH305, vorasidenib, (AG‐881), olutasidenib, (FT‐2102), AGI‐5198, AG‐120, BAY 1436032, FT‐2102, AG‐881	Mutant IDH1	2‐Hydroxyglutarate synthesis	AML	[353, 354, 355]
3	CB‐839 (Telaglenastat), BPTES, 968	GLS	Block synthesis of glutamate	CRC, TNBC, AML NSCLC, RCC	[356]
4	l‐Asparaginase, phenylbutyrate	Glutamine depletion	Uptake of glutamine from plasma	ALL	[122, 357‐360]
5	Benzylserine, l‐γ‐glutamyl‐*p*‐nitroanilide (GPNA), V‐9302, γ‐FBP	ASCT2	Inhibits transport of glutamine into cells	Multiple cancers	[361, 362]
6	Acivicin, azaserine, 6‐diazo‐5‐oxo‐l‐norleucine (DON)	Glutamine mimetics	Inhibits glutaminase	Multiple cancers	[363, 364]
7	R162, EGCG, ECG	GDH inhibitors	Blocks synthesis of α‐KG	Multiple cancers	[361, 365]
8	Amino ethoxy vinyl‐glycine (AVG), rhizobitoxine, and aminooxy acetic acid (AOA)	Aminotransferase Inhibitors	Blocks the synthesis of α‐KG	Multiple cancers	[366]
9	Seleno‐l‐methionine, HDAC, bortezomib	HIF1α	–	Multiple cancers	[367]
10	Temsirolimus, everolimus	mTOR	Inhibits mTOR	Gynecological malignancies	[368]
11	Lapatinib, iressa, ZD1839, gefitinib, tarceva (OSI‐774)	EGFR	EGFR tyrosine kinase inhibitors	NSCLC, GBM	[369, 370]

Ongoing clinical trials of small molecule inhibitors					
S. no.	Agent	Target	Mechanism	Cancer type	References
1	Sirpiglenastat (DRP‐104)	Glutamine antagonist	Targets metabolism to provide therapeutic benefits	Advanced solid tumors	[371, 372]
2	NBDHEX	Glutathione S‐transferase P1‐1 (GSTP1‐1)	It inhibits autophagy with anticancer properties.	NSCLC	[373, 374]
3	Dimethyl fumarate (DMF)	NRF2 activation	Induction of cell autophagy and the increase of antioxidant gene expression	CLC	[375, 376]
4	IACS‐010759	OXPHOS	Reduces growth and triggers apoptosis that depends on OXPHOS	AML and solid tumors	[377]
5	Nanvuranlat JPH203	l‐type amino acid transporter 1 (LAT1)	Uptake of essential amino acids in tumor cells	CLC, OSCC, and leukemia	[378, 379]
6	IPN60090	Glutaminase	Conversion of glutamine to glutamate	Advanced solid tumors	[380]
7	CPI‐613	Mitochondria	Oxidative metabolism	PDAC, AML, solid tumors, lymphoma	[381, 382]
8	AZD‐3965	MCT1	Lactate symporter	Advanced cancers	[383]
9	HCQ	Increase antiestrogen responsiveness	Inhibits autophagy	PDAC	[384]
10	AG221	Mutant IDH2	AG‐221 inhibits the conversion of KG to 2HG by binding to an allosteric site.	AML	[385]
11	PT2385	HIFα	Prevents HIF‐2α heterodimerization and DNA binding	RCC	[386]
12	PRIM‐1 Met, CP‐31398, STIMA, MIRA‐1	P53 reactivator	Activates p53	OVC, ESCC, AML, melanoma	[387, 388, 389]
13	Fasentin, STF‐31536	GLUT1	Fasentin inhibits ERK1/2 signaling and TF‐31 inhibits NAMPT and GLUT.	Multiple cancers	[390, 391]

Abbreviations: ALL, acute lymphoblastic leukemia; AML, acute myeloid leukemia; CC, colon cancer; ESCC, esophageal squamous cell carcinoma; GBM, glioblastoma; NSCLC, non‐small‐cell lung carcinoma; OSCC, oral squamous cell carcinoma; OVC, ovarian cancer; PDAC, pancreatic ductal adenocarcinoma; RCC, renal cell carcinoma; TNBC, triple‐negative breast cancer.

## 10 Therapeutic Opportunities and Clinical Trials of lncRNAs in Controlling Cancer

The therapeutic use of RNA offers several common obstacles to developing lncRNA‐based medication. These challenges render it more difficult to design treatments that target lncRNAs, including the absence of effective delivery systems, insufficient dose guidelines, and unknown adverse effects. While lncRNAs have been shown to regulate various human malignancies, the exact mechanisms through which they modulate metabolism are still largely unknown. There have been attempts to investigate lncRNA treatment in animal models using multiple techniques. Targeting lncRNAs with antisense oligonucleotides (ASOs) shows promise as a cancer treatment strategy. However, ASOs have poor membrane permeability, mainly confined to the cytoplasm, making it challenging to manipulate sub‐nuclear lncRNAs. Linking nanotechnology with ASOs may hold the potential for addressing this limitation. CRISPR/Cas9 technology has received considerable attention in cancer treatment for specific DNA alteration of targeted genes. Recent research has shown successful silencing of lncRNA‐expressing loci using CRISPR/Cas9 [350, 351, 352].

Nonetheless, there is still considerable uncertainty within the therapeutic use of CRISPR/Cas9 for lncRNA targeting in cancer therapy, as off‐target cleavage events can occur despite the system's target specificity [392, 393]. Developing more specific gene‐editing tools and techniques is crucial in this regard [394]. Viral vectors, including recombinant adenovirus, lentivirus, and retrovirus vectors, are superior RNAi transfection techniques. By neutralizing targeted RNA with exogenous double‐stranded RNA molecules like siRNAs and shRNAs, RNAi is a technique for precise gene inhibition [395, 396]. Extensive research has been conducted on applying shRNAs to target lncRNAs in cancer therapy. However, the complexity of clinical trials using viral transfection is significant, and accurate viral infection and dose control are important considerations for future applications due to species‐specific off‐target effects [396, 397]. CircRNAs and lncRNAs are being studied in the context of LC metabolism, but their specific roles are still limited. Propofol, a drug used in metabolism‐oriented treatment for LC that relies on lncRNAs or circRNAs, requires further analysis due to the inadequate clinical data on effectiveness and safety [398, 399]. Phase 2 or 3 clinical development is currently underway for miRNA‐based treatments, including anti‐miRNAs and miRNA mimics. Nevertheless, clinical practice does not now utilize circRNA‐ or lncRNA‐based therapies [221]. It is important to note that these drugs and therapies are still in the clinical trial phase, and further research is needed to determine their effectiveness and safety in treating cancer.

Further study is required to understand better the regulatory framework involved in cancer metabolism and to discover possible targets for developing cancer treatment approaches. It is anticipated that future research on the most effective treatment options for tumor patients with metabolic disorders will be influenced by the advancements achieved in mechanical analysis of lncRNA activity and metabolic signaling in recent years. But, expecting lncRNA‐targeted treatment to restore normal metabolism at this point would be unrealistic.

## 11 Conclusion and Future Perspectives

Glutamine, the most prevalent amino acid in blood and muscle, is essential to many biological functions and plays a significant part in maintaining metabolic homeostasis. While glutamine is considered a nonessential amino acid for normal cells, it becomes necessary for cancer cells to generate ATP and synthesize crucial cellular building blocks in a challenging TME. Numerous diseases, such as cancer, neurological problems, and metabolic abnormalities, are associated with elevated levels of glutamine. Nevertheless, there are insufficient data to determine a direct causative link between these diseases and aberrant glutamine levels. Although the precise processes are yet unknown, some research indicates that glutamine variations may impact the extent or progress of the disease.

Glutamate transporter and other modulator expression and function changes are linked to several diseases. Understanding whether these alterations contribute to pathogenesis or result from preexisting disease is critical. In many situations, the involvement of glutamate transporters and other modulators in pathogenesis remains unclear due to inconsistent data, potentially due to different methodologies. The metabolic process of glutaminolysis has been established as essential for multiple tumors. Neoplastic cells must absorb and metabolize external nutrients to provide energy and building blocks for unchecked tumor growth. There is growing interest in understanding how cancer cells sustain their metabolic functions by utilizing available resources. This glutamine reliance is often referred to as an Achilles’ heel of cancer, as it represents a metabolic vulnerability that can be targeted therapeutically. The complicated characteristics of cancer metabolism are highlighted by the variations in the dependency of cancer cells on metabolic sources of fuel caused by mutational heterogeneity. The regulation of glutamine metabolism largely depends on oncogenes and tumor suppressor genes. Glutamine addiction in cancer is driven by oncogenes such as c‐Myc, KRAS, and EGFR, as well as the loss of tumor suppressor genes like p53, INK4, and PTEN. Understanding the metabolic dynamics within tumors has driven the development of biologically targeted therapies that exploit these metabolic vulnerabilities. Although research suggests that both oncogene and tumor suppressor expression levels may influence glutamine metabolism in cancer cells, little is known about glutamine metabolism in cancer, especially concerning glucose metabolism.

Additionally, deregulated enzymes in glutamine metabolism contribute to cancer metabolic variations, acting as signaling molecules and activators of cancer progression factors like mTOR and autophagy. KRAS mutations may have specific effects on metabolism in tumor tissues due to their intrinsic metabolic wiring. Studying these effects requires considering the tumor suppressor background and investigating how oncogenic KRAS‐dependent metabolic changes are altered in the TME, including hypoxia, limited nutrients, and crosstalk between tumor cells and stromal cells. Myc, a common deregulated oncoprotein in cancer, is a challenging target due to its lack of enzymatic activity or structural features. Inhibiting Myc can lead to cell cycle arrest, ATP production collapse, and apoptosis. Efforts have included altering Myc DNA‐binding sites, Myc‐Max heterodimers, and transcriptional activation machinery. However, early Myc inhibitors have shown disappointing therapeutic efficacy in vivo. Non‐neoplastic settings allow for considering downstream Myc effectors, such as PDH, which could be therapeutic. Other targetable enzymes in the Myc axis have the potential for cotargeting Myc downstream genes/pathways. It found enrichment of mTOR signaling, antioxidant, and redox balance pathways in Myc target genes, providing the basis for a clinical trial using GLS and mTOR cotargeting strategy. KRAS mutations may have specific effects on metabolism in tumor tissues due to their intrinsic metabolic wiring. Studying these effects requires considering the tumor suppressor background and investigating how oncogenic KRAS‐dependent metabolic changes are altered in the TME, including hypoxia, limited nutrients, and crosstalk between tumor cells and stromal cells.

Certain cancers, like those driven by Myc, rely on glutamine, making targeting glutamine metabolism pharmacologically beneficial. Oncogenic drivers may result in tumor cells bypassing glutamine's need. Targeted inhibition of some oncogenic drivers can rewire cells to become dependent on glutamine, making targeted inhibitors potentially lethal. The field of cancer metabolism has made progress in understanding alternative fuel sources for cancers, including glutamine, which can be exploited for therapeutic purposes under specific circumstances. Targeting glutaminolysis with inhibitors of oncogenic proteins and dysregulated proteins in the glutamine pathway has shown promising therapeutic results for aggressive cancers. Clinical trials investigating small molecular inhibitors will enhance understanding of their efficacy in humans and correlate with preclinical models. As mutational heterogeneity in cancer metabolism deepens, new targets are emerging to effectively target each aspect of metabolic reprogramming. Combining dual therapy strategies may help overcome chemoresistance in glutamine‐dependent metabolic cancers.

Furthermore, ncRNAs, such as miRNAs, lncRNAs, and circRNAs, regulate glutamine addiction and metabolism. These ncRNAs modify essential hallmarks of cancer, including metabolic rewiring, allowing cancer cells to adapt to the challenging microenvironment and sustain deregulated proliferation. However, it is still unclear how exactly lncRNAs affect the hallmarks of cancer metabolic reprogramming, work, and what species they belong to. In vitro studies provided the majority of information about the metabolic activities and regulatory mechanisms of lncRNAs. Further in vivo studies using lncRNA knockdown or overexpression are required to examine their involvement in metabolic regulation. LncRNA‐based diagnostics and treatments for cancer metabolism are expected to benefit cancer patients, while clinical trials are still in the early phases. The development of lncRNA inhibitors and analytical techniques will make it possible to quickly screen for lncRNAs differently expressed in cancer and elucidate the mechanisms underlying cancer metabolism, opening the door to potential clinical applications and future approaches to cancer treatment. Altered glutamine levels are linked to various human pathologies, but limited evidence supports direct causality. Although glutamine‐targeting interventions have shown potential, they have generally lacked efficacy in vivo. The extent to which glutamine dysregulation contributes to disease development in specific mouse models is unknown due to the absence of specialized pharmacological techniques or genetically altered mouse models.

In conclusion, glutamine is a versatile molecule that plays a crucial role in various biological processes, including antioxidant defense, neurotransmission, and cellular metabolism. Alterations in glutamine levels or its metabolic pathways have been associated with multiple health disorders, ranging from neurodegenerative diseases to cancer. However, the exact causal relationships remain often unclear, complicating our understanding of how changes in glutamine metabolism contribute to these conditions. Additionally, targeting glutamine metabolism for therapeutic purposes has proven to be challenging. This difficulty arises from the complexity of glutamine's functions in the body and the need for precise interventions that do not disrupt its essential roles in normal physiological processes.

## Author Contributions

M.A.K., S.K.B., I.R.K., A.A.B., and M.A.M. wrote the manuscript and generated figures. M.A.M. and A.A.B. contributed to the concept and design and critically edited the manuscript. M.S.K., F.M.H., S.A., M.H., M.S., A.S.A.A., A.A.B., and M.A.M. performed critical revision and editing of the scientific content. All authors read and approved the final manuscript.

## Ethics Statement

Not appliable.

## Conflicts of Interest

The authors declare no conflicts of interest.

## Data Availability

Not appliable.
